# Bacterial Quorum-Sensing Signal Arrests Phytoplankton Cell Division and Impacts Virus-Induced Mortality

**DOI:** 10.1128/mSphere.00009-21

**Published:** 2021-05-12

**Authors:** Scott B. Pollara, Jamie W. Becker, Brook L. Nunn, Rene Boiteau, Daniel Repeta, Miranda C. Mudge, Grayton Downing, Davis Chase, Elizabeth L. Harvey, Kristen E. Whalen

**Affiliations:** a Department of Biology, Haverford College, Haverford, Pennsylvania, USA; b Department of Genome Sciences, University of Washington, Seattle, Washington, USA; c College of Earth, Ocean, and Atmospheric Sciences, Oregon State University, Corvallis, Oregon, USA; d Marine Chemistry and Geochemistry, Woods Hole Oceanographic Institution, Woods Hole, Massachusetts, USA; e Department of Biological Sciences, University of New Hampshire, Durham, New Hampshire, USA; University of Wisconsin—Madison

**Keywords:** HHQ, *Pseudoalteromonas*, cell cycle, phytoplankton, quorum sensing, virus-host interactions

## Abstract

Interactions between phytoplankton and heterotrophic bacteria fundamentally shape marine ecosystems by controlling primary production, structuring marine food webs, mediating carbon export, and influencing global climate. Phytoplankton-bacterium interactions are facilitated by secreted compounds; however, linking these chemical signals, their mechanisms of action, and their resultant ecological consequences remains a fundamental challenge. The bacterial quorum-sensing signal 2-heptyl-4-quinolone (HHQ) induces immediate, yet reversible, cellular stasis (no cell division or mortality) in the coccolithophore Emiliania huxleyi; however, the mechanism responsible remains unknown. Using transcriptomic and proteomic approaches in combination with diagnostic biochemical and fluorescent cell-based assays, we show that HHQ exposure leads to prolonged S-phase arrest in phytoplankton coincident with the accumulation of DNA damage and a lack of repair despite the induction of the DNA damage response (DDR). While this effect is reversible, HHQ-exposed phytoplankton were also protected from viral mortality, ascribing a new role of quorum-sensing signals in regulating multitrophic interactions. Furthermore, our data demonstrate that *in situ* measurements of HHQ coincide with areas of enhanced micro- and nanoplankton biomass. Our results suggest bacterial communication signals as emerging players that may be one of the contributing factors that help structure complex microbial communities throughout the ocean.

**IMPORTANCE** Bacteria and phytoplankton form close associations in the ocean that are driven by the exchange of chemical compounds. The bacterial signal 2-heptyl-4-quinolone (HHQ) slows phytoplankton growth; however, the mechanism responsible remains unknown. Here, we show that HHQ exposure leads to the accumulation of DNA damage in phytoplankton and prevents its repair. While this effect is reversible, HHQ-exposed phytoplankton are also relieved of viral mortality, elevating the ecological consequences of this complex interaction. Further results indicate that HHQ may target phytoplankton proteins involved in nucleotide biosynthesis and DNA repair, both of which are crucial targets for viral success. Our results support microbial cues as emerging players in marine ecosystems, providing a new mechanistic framework for how bacterial communication signals mediate interspecies and interkingdom behaviors.

## Abstract (precis)

Bacteria and phytoplankton form close associations in the ocean that are driven by the exchange of chemical compounds. The bacterial signal 2-heptyl-4-quinolone (HHQ) slows phytoplankton growth; however, the mechanism responsible remains unknown.

## INTRODUCTION

Interactions between marine phytoplankton and bacteria have been shown to fundamentally shape marine ecosystems, particularly by mediating biogeochemical cycling, regulating productivity, and trophic structure (1–3). Bacterium-phytoplankton interactions are complex, often being species specific (4) or temporally ephemeral (5), and can span the spectrum from antagonistic to beneficial (6, 7). Increasingly, it is clear that these intricate interkingdom interactions are facilitated by excreted chemical compounds that mediate a suite of processes such as nutrient transfer, primary production, and shifts in community composition. Linking chemical compound identity with a mechanism of action and ecological consequences will strengthen our understanding of how these fundamental and multifaceted interactions govern marine ecosystem function.

First discovered in marine systems 4 decades ago (8), quorum sensing (QS) is a form of microbial cell-cell communication through which marine bacteria use diffusible chemical signals to facilitate coordinated and cooperative biogeochemically important behaviors (9). Recent work finds that alkylquinolone-based QS signals can modulate interspecies behavior, suggesting that these molecules may influence cellular communication at the interkingdom level (10). In particular, the alkylquinolone QS signal 2-heptyl-4-quinolone (HHQ) functions as a messenger molecule able to modulate bacterial virulence behavior, facilitating the emergence of the pathogen Pseudomonas aeruginosa within polymicrobial communities (11, 12). Trafficking of hydrophobic alkylquinolones, including HHQ, is aided by the release of outer membrane vesicles containing micromolar concentrations of alkylquinolones that are produced by P. aeruginosa and serve as signal delivery vehicles to neighboring recipient cells (13). Purified outer membrane vesicles isolated from P. aeruginosa have also been shown to possess significant antimicrobial activity, inhibiting the growth of adjacent Gram-positive bacteria (13). Additionally, HHQ has also been implicated in antagonizing fungal biofilm formation (12); downregulating eukaryotic host immune responses via the suppression of a key transcription factor, NF-κB (10); and activating receptors found to play a role in innate immune signaling in airway epithelia (14). These findings support the influence of alkylquinolones in mediating host-microbe interactions.

Recently, HHQ was isolated from marine gammaproteobacteria (Pseudomonas sp. and *Pseudoalteromonas* sp.) and was observed to cause significant shifts in both natural phytoplankton and microbial communities (15), including species-specific static phytoplankton growth (no growth or mortality) at nanomolar concentrations (16). Static growth in phytoplankton has been observed previously, in relation to both bacterial exudates (17, 18) and nutrient stress (19–22). However, the underlying molecular mechanism(s) by which HHQ influences phytoplankton fitness and the outcomes of ecological interactions remains unknown. For example, host physiology has been demonstrated to be an integral factor in the success of viral infection of phytoplankton, with infection success and burst size being influenced by host conditions (23). However, the role that HHQ plays in mediating microbial interactions beyond phytoplankton growth alterations has yet to be investigated.

To better understand how HHQ alters molecular function and ecological interactions in marine microbes, ultrastructural observations and diagnostic biochemical assays were integrated with transcriptomic and proteomic studies to link the persistent but reversible physiological impact of nanomolar concentrations of HHQ on a model marine phytoplankton, Emiliania huxleyi. Furthermore, we examined if HHQ could disrupt virus-induced mortality in E. huxleyi, thereby ascribing a new role for bacterial quorum-sensing signals. *E. huxleyi* plays a central role in mediating ocean carbon (24) and sulfur (25) cycling; thus, the results presented here emphasize the importance of considering the ecological consequences of chemically mediated bacterium-phytoplankton interactions on global primary production and biogeochemical cycles.

## RESULTS AND DISCUSSION

### Response to HHQ exposure.

**FIG 1:**
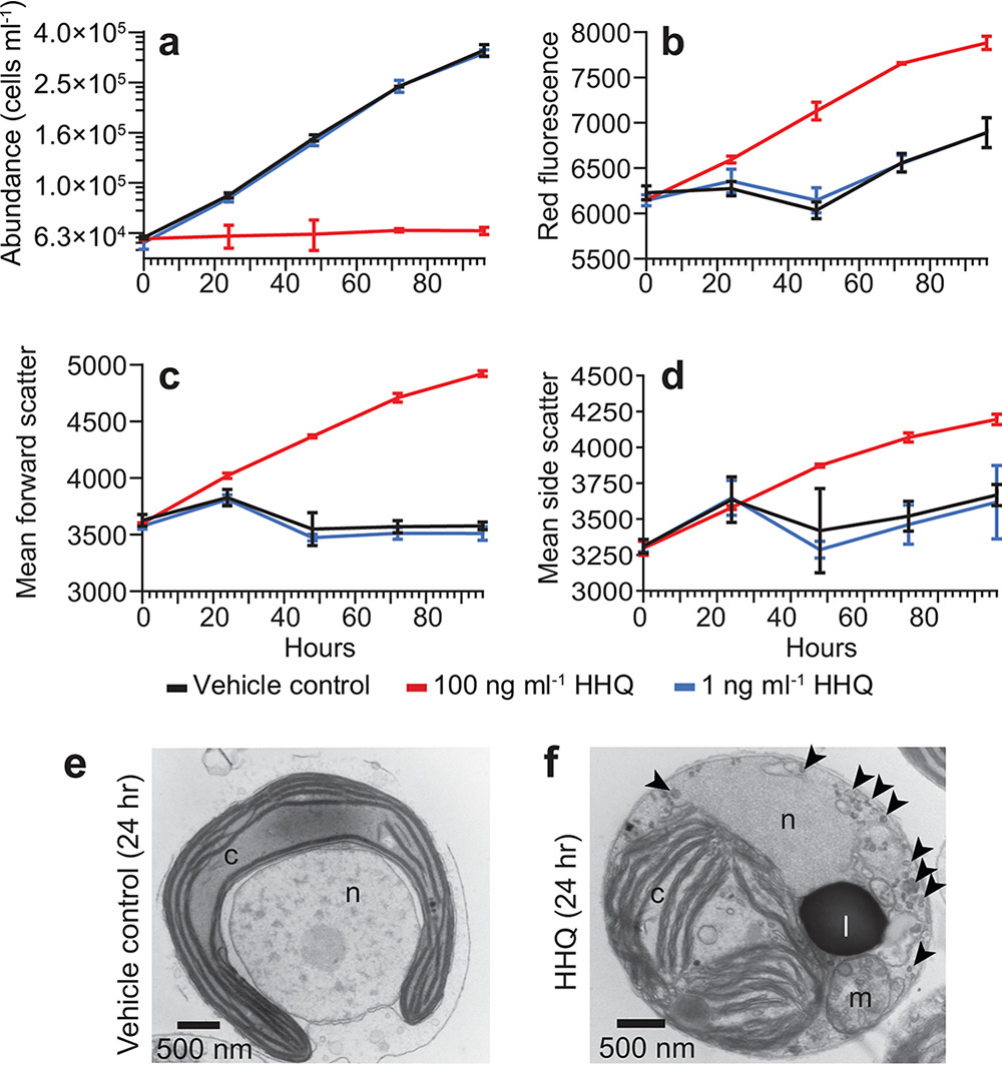
Exposure to HHQ halts cell division and alters cell morphology. (a through d) *E. huxleyi* cultures (*n* = 3) were exposed to HHQ or the vehicle control (DMSO) at the 0-h time point (*T*_0_) and monitored by flow cytometry for cell abundance (a), red fluorescence in relative fluorescence units (RFU) (695/50 nm) (a proxy for chlorophyll *a* intensity) (b), forward scatter (a proxy for cell size) (c), and side scatter (a proxy for cell granularity) (d) over 96 h. Means ± standard deviations are shown. In 100-ng ml^−1^ HHQ-exposed cells, all parameters measured were significantly different from those for the vehicle control (*P < *0.05 by repeated-measures analysis of variance). Note that in panel a, data for HHQ-treated cells at 1 ng ml^−1^ sit directly beneath data for the vehicle control (DMSO). (e and f) Transmission electron microscopy micrographs of *E. huxleyi* cells exposed to the vehicle control (DMSO) (e) or 100 ng ml^−1^ HHQ (f) for 24 h. Subcellular structures include the chloroplast (c), lipid droplet (l), mitochondria (m), nucleus (n), and vacuoles (black arrowheads).

Following 96 h of exposure to 100 ng ml^−1^ of HHQ, batch cultures of axenic *E. huxleyi* (CCMP2090) exhibited cellular stasis (no cell division or mortality) concomitant with a significant increase in forward scatter, red fluorescence, and side scatter, proxies for cell size, chlorophyll content, and cell granularity, respectively (*P* value of <0.01 for all comparisons by repeated-measures analysis of variance [ANOVAR]) (Fig. 1). The photosynthetic efficiency (*F_v_*/*F_m_*) did not change in response to long-term HHQ exposure (ANOVAR). Additionally, after only 24 h of HHQ exposure, phytoplankton cells were observed to have enlarged chloroplasts with distended thylakoid membranes containing numerous intraorganelle vesicles, abundant cytoplasmic vesicles/vacuoles, homogeneous nucleus staining lacking defined euchromatin/heterochromatin regions with disintegrated nuclear envelops, and osmium-rich puncta within and adjacent to the chloroplasts, likely indicating enhanced lipid storage (see Fig. S1 in the supplemental material). To examine if the physiological effects induced by HHQ exposure were reversible, 96-h-exposed HHQ cultures were diluted roughly ∼80-fold with f/2 medium without silica (26) to a final concentration of 1.25 ng ml^−1^ HHQ, a concentration demonstrated not to influence *E. huxleyi* growth. Cells previously exposed to HHQ showed recovery mirroring paired vehicle control cultures (ANOVAR) (Fig. S2). Taken together, HHQ-treated *E. huxleyi* cells appear to mirror previous studies in which cellular arrest has been observed in phytoplankton in response to bacterially derived chemical exposure (17, 18, 27–29) as well as nutrient limitation (20–22). In order to elucidate if the observed cellular stasis is mechanistically similar to those observed previously in the literature, we conducted cell cycle, transcriptomic, and proteomic analyses of HHQ-exposed *E. huxleyi*.

10.1128/mSphere.00009-21.2 FIG S1 Composite TEM images showing representative images of *E. huxleyi* cells exposed to the vehicle control (DMSO) (a through f) or 100 ng ml^−1^ HHQ (g through l) for 24 h. In select images, subcellular structures are labeled for identification. c, chloroplast; l, lipid droplet; m, mitochondria; n, nucleus; p, pyrenoid; black arrow, Golgi apparatus; v, vacuole; white arrowheads, vesicles. Bar = 500 nm. Download FIG S1, PDF file, 0.7 MB. Copyright © 2021 Pollara et al. 2021 Pollara et al. https://creativecommons.org/licenses/by/4.0/ This content is distributed under the terms of the Creative Commons Attribution 4.0 International license.

10.1128/mSphere.00009-21.3 FIG S2 *E. huxleyi* cultures were examined for their ability to recover following exposure to 100 ng ml^−1^ of HHQ for 96 h. Following HHQ exposure, cultures were monitored for their ability to recover by measuring the growth rate (a) (each point represents the exponential growth rate over the previous 24 h), red fluorescence in RFU (a proxy for chlorophyll *a* intensity) (b), and forward scatter (a proxy for cell size) (c). Each symbol represents the mean from three independent replicates ± the standard deviation. Significant differences between HHQ-exposed cells and the vehicle control were assessed using ANOVAR (*P < *0.05). Download FIG S2, PDF file, 0.3 MB. Copyright © 2021 Pollara et al. 2021 Pollara et al. https://creativecommons.org/licenses/by/4.0/ This content is distributed under the terms of the Creative Commons Attribution 4.0 International license.

### Evidence for S-phase arrest.

**FIG 2:**
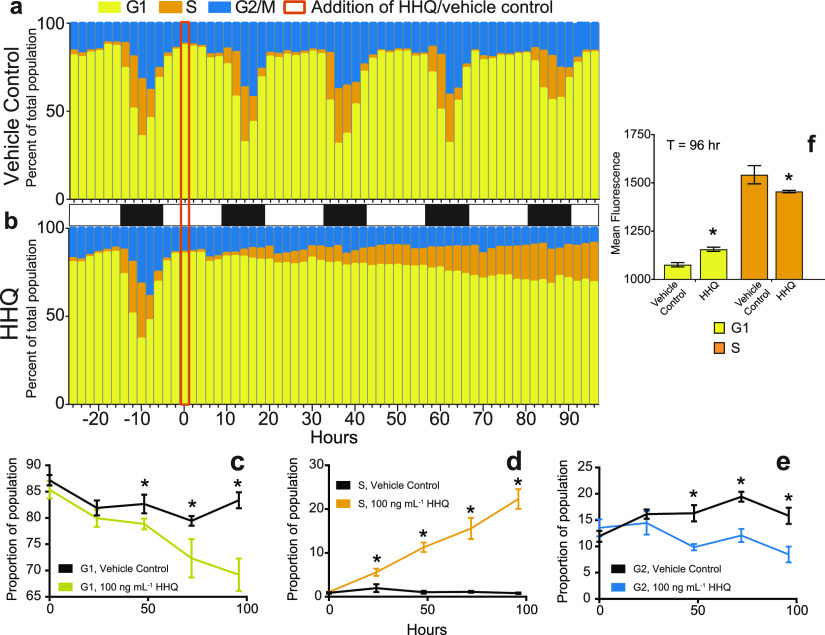
HHQ triggers stalling in S phase. (a and b) The cell cycle stage was quantified by profiling the fluorescence (575/25 nm), a proxy for DNA content, of propidium iodide-stained *E. huxleyi* cultures (*n* = 3) exposed to either the vehicle control (DMSO) (a) or 100 ng ml^−1^ HHQ (b) for 96 h. (c through e) The proportion of cells in each cell stage was determined from density plots of the distribution of cells with various DNA contents ranging from 2N (G_1_) to 4N (G_2_) at *T*_0_, *T*_24_, *T*_48_, *T*_72_, and *T*_96_. Cells with intermediate DNA content were denoted as S phase, as the genome replicated. Each plot represents the mean ± standard deviation for triplicate samples (*P < *0.05 by ANOVAR). (f) Mean fluorescences (575/25 nm) of G_1_- and S-phase cells treated with the vehicle control (DMSO) or 100 ng ml^−1^ HHQ for 96 h and stained with propidium iodide were compared via Welch’s approximate *t* test (*P < *0.01). As DNA replication occurs only in S phase, the increase in the mean fluorescence for HHQ-treated cells that fall within the G_1_ gate suggests that these cells are currently in S phase but stall early in the process of DNA synthesis and are unable to synthesize enough additional DNA to fall within the S-phase region.

The DNA content of *E. huxleyi* cells following HHQ exposure was tracked for 96 h via flow cytometry, and cells treated with HHQ ceased the typical diurnal cell cycle progression within 24 h of HHQ addition (Fig. 2). Over 96 h, the proportions of HHQ-exposed cells in both G_1_ and G_2_ phases were shown to steadily decrease, whereas the proportion of cells in S phase significantly increased (*P* > 0.01 by ANOVAR) (Fig. 2c through e). Additionally, HHQ-exposed cells found in G_1_ phase demonstrated a significantly higher DNA content per cell than their paired vehicle controls (*P* value of <0.05 by Welch’s approximate *t* test) (Fig. 2f). These results suggest that HHQ-exposed cells are attempting to duplicate their genome but are stalling in early S phase.

**FIG 3:**
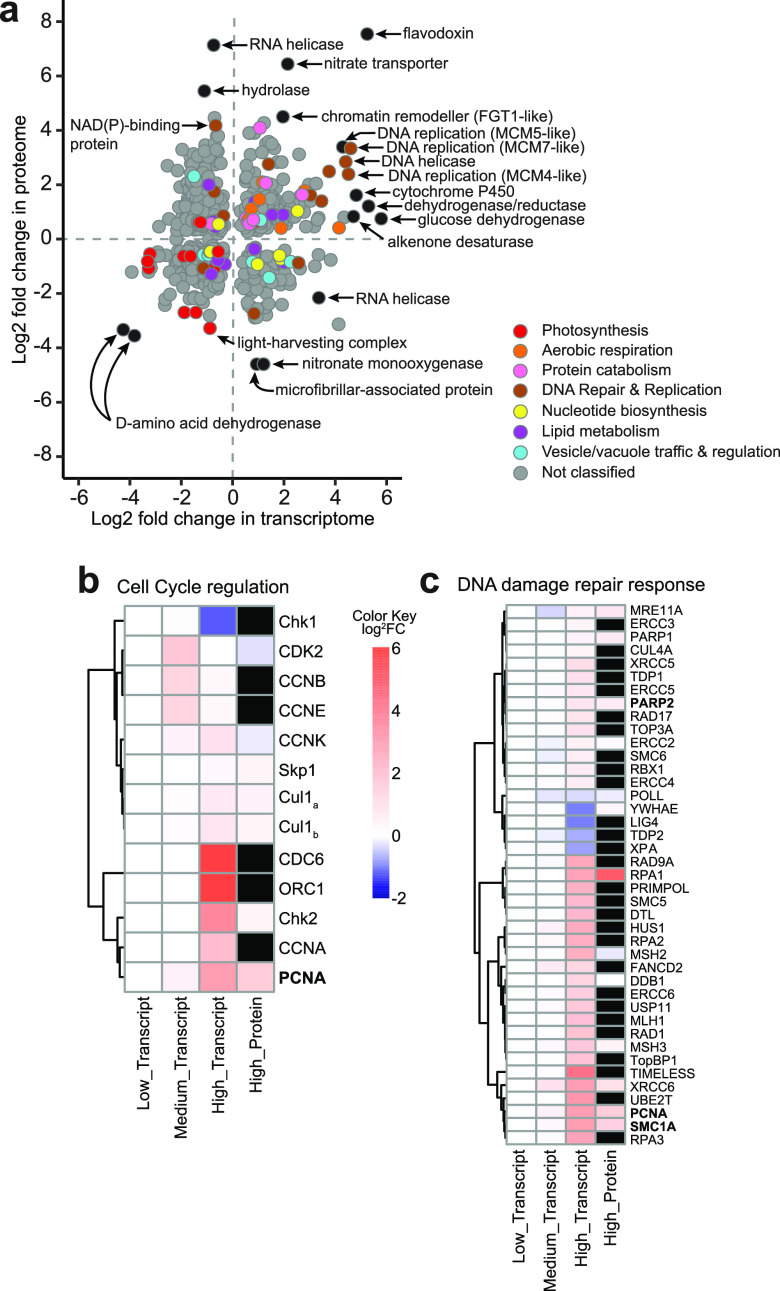
Molecular and proteomic changes as a result of HHQ exposure. (a) Comparison of log_2_ fold changes in transcript (*x* axis) and protein (*y* axis) expression from *E. huxleyi* cultures (*n* = 4) following exposure to 100 ng ml^−1^ HHQ for 72 h compared to the vehicle control (DMSO). Only shared differentially expressed transcripts (*q* value of <0.05 by a Wald test) and proteins (*q* value of <0.05 by Welch’s approximate *t* test) are shown for a total of 665 genes/proteins. Transcripts and proteins with similar functions are colored via gene ontology (GO) annotation according to the curated groupings shown in Supplemental Data File 1 at https://doi.org/10.6084/m9.figshare.14414285.v1. Genes and proteins without GO annotations or annotations outside the selected groupings are shown in gray. Selected outliers are labeled in black. (b and c) Heat maps displaying putative homologs of *E. huxleyi* protein-coding genes associated with cell cycle regulation (b) and the DNA damage repair response (c) after 72 h of HHQ exposure. Black boxes indicate proteins that were not detected in the proteomic analysis. Names in boldface type indicate those protein-coding genes found within the scatterplot in panel a. Dendrograms indicate hierarchical clustering based on the similarity of gene/protein expression levels. FC, fold change.

Whole-cell transcriptomic and proteomic analyses were performed on *E. huxleyi* cells exposed to HHQ concentrations of 1 ng ml^−1^ (low), 10 ng ml^−1^ (medium), and 100 ng ml^−1^ (high), with samples taken at 24 h (transcripts) and 72 h (transcripts and proteins) (Fig. S3). *E. huxleyi* cultures demonstrated unique transcriptomic and proteomic profiles in response to each HHQ concentration, with the greatest numbers of differentially expressed genes being found in higher-HHQ treatments, compared to the dimethyl sulfoxide (DMSO) vehicle control (Table 1; Fig. S3a and b). After 24 h of HHQ exposure, 39.8% of transcripts in high-HHQ samples were differentially expressed relative to the DMSO vehicle controls (*q* value of <0.05 by a Wald test) (Table 1). Similarly, after 72 h of exposure, replicate high-HHQ samples continued to appear distinct from the DMSO vehicle control samples (Fig. S3a and b), with 37.6% of transcripts (*q* value of <0.05 by a Wald test) and 15.9% of proteins (*q* value of <0.05 by Welch’s approximate *t* test) significantly changing in relative abundance and abundance, respectively (Table 1). When examined together, a total of 665 genes and corresponding proteins were found to significantly change in abundance at 72 h under high-HHQ treatment relative to the vehicle control (Fig. 3) (see Supplemental Data File 1 at https://doi.org/10.6084/m9.figshare.14414285.v1). In general, processes associated with DNA replication and repair, aerobic respiration, and protein catabolism yielded higher relative transcript and protein abundances under high-HHQ treatment, while photosynthetic components/processes were detected at lower relative transcript and protein abundances (Fig. 3a) (see Supplemental Data File 1 at https://doi.org/10.6084/m9.figshare.14414285.v1). Far fewer genes and proteins were found to be differentially expressed in the low- and medium-HHQ treatments (Table 1), which is likely related to the observed recovery of cell growth in these treatments (Fig. S3c). The growth of cells exposed to low HHQ concentrations was nearly identical to that of the DMSO control throughout the experiment. By 72 h, no genes or proteins in the low-HHQ treatment were differentially expressed compared to the control. While the medium-HHQ treatment demonstrated some growth inhibition over the first 24 h, by 72 h, the population had largely recovered (Fig. S3c). For cell populations exposed to medium HHQ concentrations at 24 h, transcripts related to cell cycle progression, cytoskeletal regulation, and mitosis demonstrated increased relative abundances compared to the control (see Supplemental Data File 1 at https://doi.org/10.6084/m9.figshare.14414285.v1). By 72 h, there was no clear trend observed in functions related to differentially expressed transcripts in the medium-HHQ samples. For the purposes of elucidating the molecular target(s) of HHQ, we therefore focused our efforts on the analysis of the high-HHQ transcriptomic and proteomic data.

**TABLE 1:** Summary of differentially expressed transcripts and proteins following HHQ exposure^*a*^

HHQ concn (ng/ml)	No. of transcripts or proteins					
	24 h			72 h		
	Upregulated	Downregulated	Unchanged	Upregulated	Downregulated	Unchanged
Transcripts						
1	13	20	31,476	0	0	31,549
10	2,702	1,990	26,817	382	159	31,008
100	5,948	6,605	18,956	6,166	5,698	19,685

Proteins						
1	NA	NA	NA	0	0	5,528
10	NA	NA	NA	3	0	5,525
100	NA	NA	NA	628	375	4,525

a Expression with each HHQ treatment was compared to expression with the vehicle control (DMSO) treatment (*q* value of <0.05 by Welch’s approximate *t* test). NA, not applicable.

10.1128/mSphere.00009-21.4 FIG S3 (a) Principal-component analysis performed on the regularized-logarithm-transformed transcriptomic read count matrix. Each point represents an experimental sample, with point color indicating the treatment and shape indicating the time point. The first component explains 39% of the variance, and the second component explains 20%. (b) Principal-component analysis performed on the logarithm-transformed proteomic data matrix (protein summed peptide peak area values + 1). Each point represents an experimental sample, with point color indicating the treatment. The first component explains 29.8% of the variance, and the second component explains 16.2%. Note that protein was collected only at the 72-h time point. (c) Growth curve of *E. huxleyi* (CCMP2090 [axenic]) cultures exposed to 1, 10, and 100 ng ml^−1^ HHQ and sampled for RNA (at 24 and 72 h) and protein (at 72 h). The arrow indicates the time of HHQ addition. Download FIG S3, PDF file, 0.2 MB. Copyright © 2021 Pollara et al. 2021 Pollara et al. https://creativecommons.org/licenses/by/4.0/ This content is distributed under the terms of the Creative Commons Attribution 4.0 International license.

Indeed, at the physiological level, the response of *E. huxleyi* to HHQ parallels phosphorus (P) limitation in phytoplankton (i.e., S/G_2_-phase arrest, decreased growth rate, and increased chlorophyll content, forward scatter, and side scatter) (19–21, 30). However, the canonical response in P-limited cells of the upregulation of both alkaline phosphatase and phosphodiesterases (31–33) was not observed in cells exposed to HHQ, nor did we see a significant induction of acid phosphatases, pyrophosphatase, phosphorus transporters, or ATP-sulfurylase enzymes known to be induced following P limitation in HHQ-exposed cells, indicating the lack of phosphorus stress (see Supplemental Data File 1 at https://doi.org/10.6084/m9.figshare.14414285.v1). Therefore, while the patterns of cell cycle arrest are similar between HHQ-treated *E. huxleyi* and nutrient limitation, the underlying mechanisms are distinct.

In phytoplankton, cellular arrest is often accompanied by the induction of autocatalytic or programmed cell death (PCD) responses such as increased reactive oxygen production or caspase-like activity (34), and previous findings in mammalian cells indicate that HHQ has the ability to activate PCD pathways (35). However, no evidence of PCD/apoptosis was observed in HHQ-exposed *E. huxleyi* cells using a series of diagnostic fluorescence assays (i.e., membrane permeabilization, caspase activity, and reactive oxygen species [ROS] and nitrous oxide [NO] production) (Fig. S4). Additionally, no transcripts or proteins associated with PCD increased in abundance with exposure to HHQ (see Supplemental Data File 1 at https://doi.org/10.6084/m9.figshare.14414285.v1). The lack of PCD induction in HHQ-exposed cells may stem from *E. huxleyi*’s arrest in early S phase (Fig. 2d), as cellular arrest during S phase does not induce apoptotic pathways but rather curtails DNA replication, thereby dramatically extending the cell cycle (36). The transcriptomic profile of HHQ-exposed cells demonstrates an increased relative abundance of canonical transcripts facilitating the G_1_/S transition, including cell division control protein 6 (CDC6), origin recognition complex subunit 1 (ORC1), and cyclins A, B, E, and K (Fig. 3b). Moreover, significant increases in relative transcript abundances of DNA replication fork machinery (i.e., DNA polymerases α, ε, and δ; DNA primase; replication protein A; topoisomerases [TOPO]; the minichromosomal maintenance complex; proliferating cell nuclear antigen; and replication factor C) (Fig. 3c) 72 h after HHQ exposure suggest an intent to replicate DNA, a hallmark of S phase (37). Yet despite this observed induction of DNA replication machinery, DNA synthesis was severely curtailed following HHQ exposure (Fig. 2), suggesting that HHQ exposure interferes with the ability of *E. huxleyi* cells to correctly complete the DNA replication process.

10.1128/mSphere.00009-21.5 FIG S4 Diagnostic biochemical assays for caspase activity from whole-cell lysates or purified caspase enzyme (*n* = 3) (comparison of the whole-cell lysate at 72 h of DMSO treatment versus the whole-cell lysate at 72 h of HHQ treatment [*P* value of <0.05 by Welch’s approximate *t* test {no significance observed}]) (a), the presence of active caspase proteases *in vivo* (CaspACE) (b), reactive nitrogen species (DAF-FM diacetate) (c), reactive oxygen species (CM-H_2_DCFDA) from DMSO- or HHQ-exposed cultures from 1 to 72 h posttreatment (d), reactive oxygen species (CM-H_2_DCFDA) from 72-h DMSO- or HHQ-exposed cultures that were spiked with the algicide tetrabromopyrrole (TBP) (positive control) for 1 or 2 h (e), apoptosis (Image-iT Dead stain) from DMSO- or HHQ-exposed cultures from 1 to 72 h posttreatment (f), apoptosis (Image-iT Dead stain) from 72-h DMSO- or HHQ-exposed cultures that were spiked with TBP (positive control) for 4.5 h (g), and mitochondrial membrane integrity (MitoHealth stain) (h). Bars represent the means ± standard deviations from triplicate readings. Significance in panels b through d, f, and h was based on ANOVAR followed by Dunnett’s multiple-comparison test (*P* value of <0.05). Significance in panels e and g was based on Student’s *t* test (*P* value of <0.05). Cells exposed to HHQ were significantly different from the control only using MitoHealth stain after 24 h of exposure. However, the two treatments were not significantly different from one another at all subsequent time points. Download FIG S4, PDF file, 0.3 MB. Copyright © 2021 Pollara et al. 2021 Pollara et al. https://creativecommons.org/licenses/by/4.0/ This content is distributed under the terms of the Creative Commons Attribution 4.0 International license.

Disruption of DNA replication induces DNA damage response (DDR) pathways, activating effector kinases such as Chk1 and Chk2 necessary for halting DNA synthesis and the induction of cell cycle arrest to allow time for repair (38). We observed transcripts for Chk1 and Chk2 to be differentially expressed under HHQ treatment (Fig. 3b). Furthermore, a significant decrease in relative histone transcript and protein abundances, a hallmark of DNA synthesis disruption, was observed (see Supplemental Data File 1 at https://doi.org/10.6084/m9.figshare.14414285.v1) following HHQ exposure. As DNA replication and histone production are coupled, cells experiencing DNA replication stress will remove histone transcripts (39).

### Possible protein targets of HHQ.

During S phase, a cell must tightly regulate the availability of nucleotides to ensure faithful DNA replication (40). Therefore, S-phase cells rely on *de novo* nucleotide synthesis pathways to produce enough materials for complete genome replication (41). Several transcripts and proteins involved in *de novo* purine (amidophosphoribosyltransferase, trifunctional purine biosynthetic protein adenosine 3, phosphoribosylformylglycinamidine synthase, bifunctional purine biosynthesis protein, adenylosuccinate synthase, IMP dehydrogenase, and GMP synthase) and pyrimidine (carbamoyl phosphate synthase II, aspartate carbamoyltransferase, and CTP synthases) nucleotide synthesis increased in abundance with HHQ exposure (see Supplemental Data File 1 at https://doi.org/10.6084/m9.figshare.14414285.v1). Increased nucleotide synthesis may indicate the need to produce the necessary materials to replenish nucleotide pools during replication. However, only partial replication of the *E. huxleyi* genome following HHQ exposure was observed (Fig. 2), suggesting that HHQ may disrupt nucleotide production, thereby limiting nucleotide availability.

**FIG 4:**
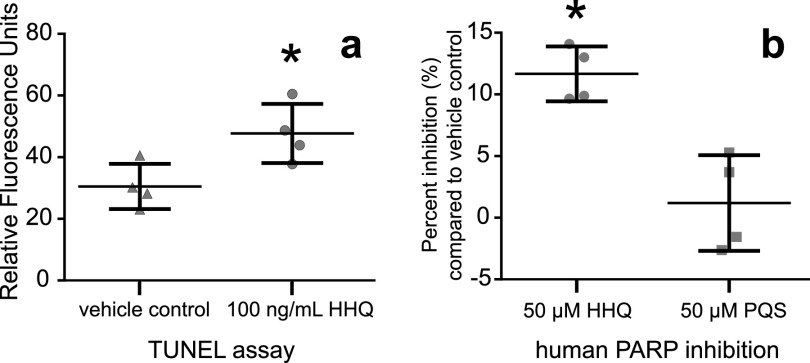
Exposure to HHQ leads to cellular DNA damage and inhibition of human PARP. (a) Cultures (*n* = 4) of *E. huxleyi* were exposed to 100 ng ml^−1^ HHQ or the vehicle control (DMSO) for 46 h before pigments were removed and cells were stained using an *in vivo* TUNEL assay to detect the presence of DNA ends, a proxy for DNA breaks. (b) Inhibition of the human PARP-1 enzyme by 50 μM HHQ and 2-heptyl-3-hydroxy-4(1H)-quinolone (PQS). Percent PARP inhibition was measured using the PARP universal colorimetric assay kit (R&D Systems). The absorbance values for quadruplicate wells containing HHQ or PQS were compared to those of the vehicle control, and this ratio was subtracted from 100% to determine PARP inhibition. Points represent individual replicates. Asterisks indicate a significant difference between the treatment and the vehicle control (*P* value of <0.05 by Welch’s approximate *t* test).

Select alkylquinolones are known to inhibit a key rate-limiting enzyme directly involved in pyrimidine synthesis, dihydroorotate dehydrogenase (DHODH) (42). DHODH inhibition in eukaryotes may induce intra-S-phase arrest due to severely diminished cellular nucleotide pools that can disrupt DNA replication, stall replication forks, and increase the frequency of genomic DNA lesions, including strand breaks, during S phase (43, 44). Indeed, after 46 h of HHQ exposure, a significant increase in DNA strand breaks was observed in culture (*P *= 0.032 by Welch’s approximate *t* test) (Fig. 4a) and was not observed when HHQ was directly exposed to genomic *E. huxleyi* or lambda DNA (Fig. S5). This indicates that DNA strand breaks are not caused directly by HHQ but are caused indirectly through other mechanisms. It has been previously observed that following the induction of DNA damage during S phase, cells will enter intra-S-phase arrest that drastically decreases the rate of DNA replication to allow the DDR to resolve any DNA lesions (36). With the exception of preliminary work in Chlamydomonas reinhardtii and dinoflagellates, the DDR has not been well characterized in phytoplankton (45, 46). Of the 57 mammalian DDR protein homologs in the *E. huxleyi* genome (E value of ≤10^−20^), 41 were significantly differentially expressed (at the transcript and/or protein level), of which 37 increased in relative abundance at 72 h under high-HHQ exposure (Fig. 3c), indicating that the cell is attempting to repair DNA lesions. However, DNA damage induced by the inhibition of DHODH is known to activate apoptotic pathways through the hyperactivation of the DDR (47). No apoptotic pathway activation was observed with HHQ exposure, suggesting that the DDR itself may also be impacted by HHQ.

10.1128/mSphere.00009-21.6 FIG S5 HHQ does not directly lead to DNA strand breaks. Lambda DNA and genomic DNA isolated from *E. huxleyi* CCMP2090 were incubated with 100 ng ml^−1^ HHQ or the volumetric equivalent of DMSO for 24 h at 18°C, and the presence of DNA strand breaks was assessed by an agarose gel stained with ethidium bromide. Download FIG S5, PDF file, 0.6 MB. Copyright © 2021 Pollara et al. 2021 Pollara et al. https://creativecommons.org/licenses/by/4.0/ This content is distributed under the terms of the Creative Commons Attribution 4.0 International license.

A master regulator of the DDR involved in chromatin remodeling, nucleolar structure, and genome stability is poly(ADP-ribose) polymerase (PARP) (48). PARP binds to sites of DNA damage and stalls replication forks, producing negatively charged ADP-ribose polymer scaffolds that attract repair proteins (49). PARP homologs in *E. huxleyi* were found to be increased in both relative transcript abundance and protein abundance under HHQ treatment (Fig. 3c). Under high levels of DNA damage or if repair mechanisms are compromised, PARP can become overactivated and deplete cellular NAD^+^ and ATP pools, thereby initiating apoptotic pathways (50). However, no apoptotic activity was observed in *E. huxleyi* cells following HHQ exposure (Fig. S4), indicating that HHQ may inhibit PARP activity. Indeed, HHQ was found to significantly inhibit human PARP activity (*P* = 0.0002 by Welch’s approximate *t* test) (Fig. 4b), while a closely related alkylquinolone, 2-heptyl-3-hydroxy-4(1H)-quinolone (PQS), did not possess PARP-inhibitory activity, nor did it impact *E. huxleyi* growth (Fig. 4b and Fig. S6a). Interestingly, the genomes of phytoplankton species unaffected by HHQ (16) did not reveal the presence of any PARP homologs, further implicating PARPs in the response of phytoplankton to HHQ.

10.1128/mSphere.00009-21.7 FIG S6 Impact of alkylquinolones on phytoplankton growth and protein homology modeling. (a) Effect of PQS on *E. huxleyi* growth. Shown is a dose-response curve of *E. huxleyi* (strain CCMP2090 [axenic]) in response to PQS. Each symbol represents the mean from three independent replicates ± the standard deviation. Protein homology modeling of the *E. huxleyi* PARP-1-like homolog (GenBank accession number XP_005783504.1) to the crystal structure of human PARP-1 (PDB accession number 2RD6) with the PARP inhibitor veliparib was performed. (b) The sequence of the *E. huxleyi* PARP-like homolog (GenBank accession number XP_005783504.1) was aligned to the sequence of human PARP-1 (PDB accession number 2RD6). Two key amino acid residues (red), Tyr and Glu, are strictly conserved between human PARP and the *E. huxleyi* protein. (c and d) The active site of the structure under PDB accession number 2RD6 shows the binding of the inhibitor veliparib. Regions in the structure under PDB accession number 2RD6 with the greatest homology to the *E. huxleyi* PARP-like homolog are shown in pink, with the remainder of the protein chain in green. The region comprising the active site for small-molecule binding is highly conserved between the two proteins, with two key binding interactions observed: the nearly coplanar arrangement of a tyrosine side chain phenyl to the inhibitor and a water-mediated hydrogen bond of the basic nitrogen atom of the inhibitor to a glutamate side chain carboxyl. Download FIG S6, PDF file, 0.9 MB. Copyright © 2021 Pollara et al. 2021 Pollara et al. https://creativecommons.org/licenses/by/4.0/ This content is distributed under the terms of the Creative Commons Attribution 4.0 International license.

Inhibition of PARP activity in the presence of DNA damage drastically reduces the effectiveness of the DDR and is known to induce cellular arrest in the S phase (51). Together, our observations of prolonged S-phase arrest (Fig. 2), the upregulation of the DDR in HHQ-exposed cultures (Fig. 3c), the conserved nature of the mammalian and *E. huxleyi* PARP catalytic sites (Fig. S6b through d), and the chemical structural similarities of HHQ to known inhibitors of both PARP and DHODH with core benzimidazole moieties (52) collectively suggest that HHQ may function simultaneously to inhibit both PARP and DHODH activity in *E. huxleyi*. Additional experiments using *E. huxleyi* enzymes are needed to fully characterize whether PARP and DHODH are molecular targets of HHQ.

### HHQ impacts on energy production.

To facilitate DNA synthesis and repair, the cell requires large ATP pools (53). In HHQ-exposed cells, the increased relative transcript abundance of enzymes in the tricarboxylic acid (TCA) cycle (i.e., isocitrate dehydrogenase, α-ketoglutarate dehydrogenase, succinate dehydrogenase, fumarase, and malate dehydrogenase) (see Supplemental Data File 1 at https://doi.org/10.6084/m9.figshare.14414285.v1) may signal the overproduction of reducing equivalents for ATP production via oxidative phosphorylation. Additionally, the increase in the relative transcript abundance of metabolic efficiency controllers, sirtuin-like deacetylases (54), observed following HHQ treatment (see Supplemental Data File 1 at https://doi.org/10.6084/m9.figshare.14414285.v1) may be a direct result of PARP inhibition. Sirtuins compete with PARPs for NAD^+^, and the expression of deacetylases is dependent on NAD^+^ availability (55). PARP inhibition is known to drastically increase cellular NAD^+^ pools, thereby promoting sirtuin expression and activity (56). Increased sirtuin activity in HHQ-exposed cells may also explain the increase in the relative transcript abundance of manganese superoxide dismutase (Mn-SOD) (see Supplemental Data File 1 at https://doi.org/10.6084/m9.figshare.14414285.v1), an antioxidant enzyme that protects the cell from ROS-induced damage, as sirtuins are known to induce the production of Mn-SOD proteins (57). Finally, increased relative transcript abundance of the tryptophan-mediated *de novo* NAD^+^ synthesis pathway was also observed, potentially in an attempt to increase NAD^+^ availability (see Supplemental Data File 1 at https://doi.org/10.6084/m9.figshare.14414285.v1). Taken together, these results suggest that HHQ exposure promotes increased energy production in *E. huxleyi*, which can fuel various cellular biosynthesis and repair pathways while staving off the induction of PCD.

Increased cellular demand for ATP would necessitate the induction of glycolytic enzymes. However, following HHQ treatment, there was a significant decrease in the relative transcript abundance of hexokinase (see Supplemental Data File 1 at https://doi.org/10.6084/m9.figshare.14414285.v1), the first step in glycolysis, consistent with previous work demonstrating that alkylquinolones suppress the induction of this glycolytic enzyme through direct targeting of transcription factor hypoxia-inducible factor 1 (HIF-1) protein degradation via proteasomal pathways (58). Furthermore, we observed a shift to the Entner-Doudoroff glycolytic pathway in HHQ-treated cells (see Supplemental Data File 1 at https://doi.org/10.6084/m9.figshare.14414285.v1), which can conserve amino acid resources due to a low protein demand in comparison to other pathways (59). Moreover, we observed increases in relative transcript abundances leading to the production of aspartate (i.e., the TCA cycle, the aspartate-arginosuccinate shunt, glutamic oxaloacetic transaminase [GOT], and C_4_-like photosynthesis) in parallel with decreases in transcripts for aspartate utilization pathways, with the exception of nucleotide synthesis (see Supplemental Data File 1 at https://doi.org/10.6084/m9.figshare.14414285.v1). Aspartate is known to rescue cells from S-phase arrest by fueling *de novo* nucleotide synthesis (60).

### HHQ impacts on photosynthesis and redox.

HHQ-induced cell cycle arrest in *E. huxleyi* did not significantly alter the photosynthetic energy conversion efficiency; however, the majority of light-harvesting complexes and transcripts of the Calvin cycle decreased in relative abundance under HHQ exposure (Fig. 3a). These findings parallel those described previously for the diatom Phaeodactylum tricornutum undergoing chemically mediated cell cycle arrest (61). In plants, the coordinated downregulation of transcripts involved in photosynthesis, electron transport, and the Calvin cycle is thought to allow for the reallocation of resources toward defense against bacterial and viral pathogens (62). However, a decrease in transcript abundance does not always correlate with a loss of protein function, as photosynthetic proteins have a long functional half-life in the cell, with the exception of ferredoxin (Fd) and ferredoxin NADP^+^ oxidoreductase (FNR), both of which are involved in maintaining the cellular redox state following pathogen infection (62). Together, both ferredoxin and the isofunctional flavodoxin (Fld) participate in electron shuttling, preventing electron misrouting that can lead to ROS accumulation and restoring chloroplast redox homeostasis under environmental stress (63). Indeed, the genes and proteins with the most significant differential expression levels under HHQ exposure in *E. huxleyi* were Fd (58-fold increase in transcript and 3-fold increase in protein abundances), FNR (85-fold increase in transcript abundance), and Fld (38-fold increase in transcript and 186-fold increase in protein abundances) (Fig. 3a) (see Supplemental Data File 1 at https://doi.org/10.6084/m9.figshare.14414285.v1), which may explain the observed lack of ROS production (Fig. S4). Additional reduction systems, including FAD/NAD(P)-binding oxidoreductase, ferredoxin nitrite reductase (Fd-NR), and glutathione reductase (GR), in HHQ-treated *E. huxleyi* cells were also significantly induced, which could ameliorate NADPH buildup (see Supplemental Data File 1 at https://doi.org/10.6084/m9.figshare.14414285.v1). Moreover, increased relative expression of vitamin B_6_ (VitB_6_) transcripts following HHQ treatment could protect against oxidative stress in chloroplasts (64), while increased relative expression levels of transcripts encoding proline oxidase (POX), pyrroline-5-carboxylate reductase (P5CR), and manganese superoxide dismutase (Mn-SOD) could explain the lack of mitochondrial ROS toxicity (Fig. S4) (see Supplemental Data File 1 at https://doi.org/10.6084/m9.figshare.14414285.v1). Together, these results suggest that HHQ-exposed *E. huxleyi* uniformly decreased the relative abundance of photosynthetic gene transcripts in support of a coordinated induction of defense responses aimed at maintaining cellular redox homeostasis without debilitating photosynthetic capacity.

### Consequences of HHQ-induced cellular stasis.

**FIG 5:**
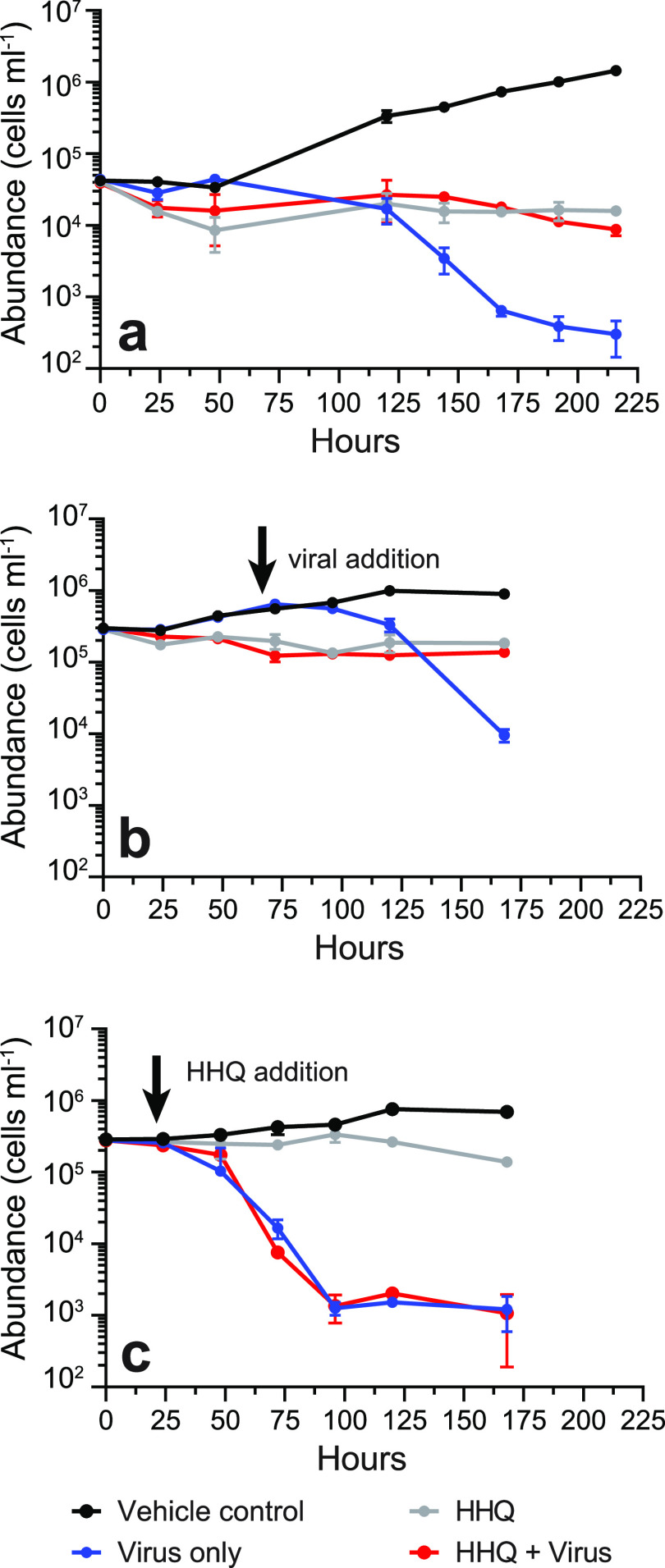
HHQ can inhibit *E. huxleyi* virus-induced mortality. The abundance (cells per milliliter) of *E. huxleyi* over time (hours) after being exposed to either the vehicle control (DMSO), HHQ (100 ng ml^−1^), EhV 207, or HHQ plus EhV 207 (MOI = 80) was determined. (a) HHQ and the virus were added together. (b) HHQ was added at *T*_0_, and the virus was added after 72 h. (c) Virus was added at *T*_0_, and HHQ addition was delayed for 24 h (*n* = 3 for each treatment in each experiment). Means ± standard deviations are shown.

**FIG 6:**
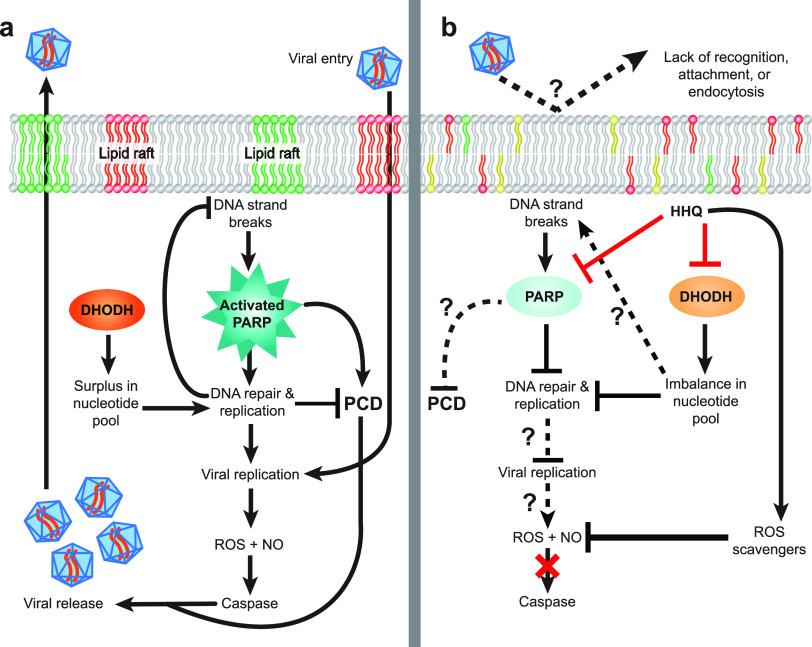
Proposed model for the role of HHQ in influencing viral success in *E. huxleyi*. (a) During the infection of a phytoplankton cell, viruses are recognized via specific surface receptors and will enter the cell via endocytosis through distinct lipid rafts. Once inside the cell, the virus hijacks host replication machinery to produce additional viral particles. This replication is dependent on functional *de novo* nucleotide synthesis enzymes, such as dihydroorotate dehydrogenase (DHODH), to provide the cell with sufficient nucleotide materials. Likewise, functional DNA repair, often mediated by poly(ADP-ribose) polymerase (PARP), is necessary to ensure that replication can continue. Successful viral replication then generates intracellular reactive oxygen species (ROS) and nitric oxide (NO) signaling, which in turn activates caspase proteases, allowing the release of replicated viral particles via programmed cell death (PCD)-induced cell lysis. (b) In HHQ-exposed phytoplankton cells, virus-induced mortality was not observed, but the mechanism by which HHQ impacts viral cycling remains unclear. HHQ may directly inhibit (shown as red lines) the activity of DHODH and PARP, which would prevent the production of viral particles via the collapse of DNA replication machinery. HHQ may also indirectly impact parts of the virus cycle (shown as dotted lines) by changing host physiology to disrupt recognition, nucleotide production, ROS production, caspase activation, or PCD.

Given that viral replication requires the hijacking of host replication machinery and HHQ exposure inhibited DNA replication and repair in *E. huxleyi*, the impact of HHQ on host-virus dynamics was investigated. When *E. huxleyi* cells were exposed to HHQ and Emiliania huxleyi virus (EhV) strain 207 concurrently, virus-induced cellular death was significantly reduced (*P* value of <0.0001 by ANOVAR) (Fig. 5a). This outcome was observed regardless of whether viruses were added simultaneously with HHQ (Fig. 5a) or 72 h after HHQ treatment (Fig. 5b). However, if HHQ addition was delayed even by 24 h, virus-induced mortality occurred in *E. huxleyi* (Fig. 5c). These results indicate the possibility that HHQ exposure early in viral infection critically impacts the effectiveness of the virus. There are numerous mechanisms by which HHQ may inhibit virus-induced mortality of *E. huxleyi* (Fig. 6). For example, HHQ may impact the entry of the virus into the cell. Significant morphological restructuring occurred following 24 h of HHQ exposure, which may prevent viral recognition, attachment, and/or endocytosis. Previous work has demonstrated that within 24 h of *E. huxleyi* viral infection, the virus requires the induction of host DNA replication machinery (65). Thus, HHQ may either inhibit the virus’ ability to manipulate DNA replication or acquire necessary nucleotides for transcription, thereby stalling infection success. HHQ may also stall the induction of ROS production, which has been demonstrated previously to be necessary for successful *E. huxleyi* viral infection (66). In the transcriptomic and proteomic data presented here, significant upregulation of a variety of antioxidants, including Fd, FNR, Fld, Fd-NR, GR, Mn-SOD, POX, and VitB_6_, may counteract virus-induced remodeling of the host antioxidant network essential for viral replication. Likewise, the expression and activation of caspase and metacaspase proteases during infection are critical for enabling virus-induced lysis in *E. huxleyi* (67). However, these proteases were not upregulated and did not show activity in HHQ-exposed cells (Fig. S4) (see Supplemental Data File 1 at https://doi.org/10.6084/m9.figshare.14414285.v1), further suggesting that HHQ exposure may disrupt these critical processes in viral infection. Attenuation of viral mortality would theoretically permit increased survival of phytoplankton and allow bacteria to continue to take advantage of coordinated nutrient exchange, common between bacteria and phytoplankton (68). Thus, the impacts of HHQ exposure on phytoplankton may have ecological consequences beyond shifts in algal physiology, to impacts on large-scale biogeochemical cycles.

### Summary.

Our laboratory findings demonstrate that a quorum-sensing signal produced by a marine bacterium significantly, but reversibly, leads to DNA lesions and cell cycle arrest in a eukaryotic phytoplankter, which can influence interkingdom virus-host interactions. In the eastern tropical South Pacific, >1-ng liter^−1^ surface concentrations of HHQ were found to correlate with areas of enhanced phytoplankton biomass (Fig. S7). These low concentrations of HHQ in bulk seawater are not surprising, as *N*-acyl homoserine lactones (69), vitamins (70), and other highly labile, trafficked compounds fundamental for growth and signaling are often found in low concentrations in bulk seawater. We anticipate that the primary abiotic sink for HHQ will be photooxidation by sunlight; however, the rate of photooxidation in seawater will strongly depend on a myriad of factors (e.g., depth, day length, and dissolved organic matter [DOM] concentrations, etc.). Previous work showed that the half-life of quinoline, the parent compound of HHQ, varied between 8 and 53 days using solar simulations (71). Furthermore, these measured bulk concentrations likely do not represent the effective concentration that a marine microbial cell would experience in the phycosphere (72).

10.1128/mSphere.00009-21.8 FIG S7 Detection of HHQ in the marine environment. (a) Cruise track of the U.S. GEOTRACES GP16 cruise in 2013 in the eastern southern tropical Pacific Ocean. (b) Composite figure showing the detection of HHQ in environmental samples. Gray bars indicate the concentrations of HHQ from six stations along the cruise track. Circles indicate the percent relative abundances of picoplankton (yellow) and micro- and nanoplankton (red). Previously, HHQ was isolated from laboratory cultures of both marine *Pseudoalteromonas* and Pseudomonas spp. (c and d) Extracted ion chromatograms for HHQ ([M + H]^+^ = *m/z* 244.17) detected by high-resolution (450K) orbitrap liquid chromatography-electrospray ionization mass spectrometry (LC-ESIMS) for the standard (c) and environmental (d) samples. (e) HHQ confirmation via tandem mass spectrometry (MS/MS) analysis in environmental samples compared to an authentic standard. High-mass-accuracy high-energy collisional dissociation (HCD) fragmentation spectra (35% collision energy) of the [M + H]^+^ ion were collected at 38 min. Download FIG S7, PDF file, 0.4 MB. Copyright © 2021 Pollara et al. 2021 Pollara et al. https://creativecommons.org/licenses/by/4.0/ This content is distributed under the terms of the Creative Commons Attribution 4.0 International license.

Previous work has demonstrated that HHQ can significantly alter natural microbial community composition and growth rates (15), and here, we find that detectable *in situ* HHQ concentrations correlated with enhanced phytoplankton biomass. Together, these findings suggest that alkylquinolone signaling may play a significant role in structuring complex microbial communities, ultimately influencing primary production and biogeochemical cycles. In addition, our findings highlight the functional duality of bacterial cues that serve as diffusive messengers used as a communication tool in microbial communities but also as chemical mediators of marine microbial interactions.

## MATERIALS AND METHODS

### General cultivation conditions.

For all experiments, axenic *Emiliania huxleyi* (CCMP2090, non-lith forming) (from the National Center for Marine Algae and Microbiota, East Boothbay, ME) was grown in natural seawater-based f/2 medium without silica (26). Cultures were maintained on a 14-h/10-h light (80 ± 5 μmol photons m^−2^ s^−1^)/dark cycle at 18°C, with a salinity of 35. These conditions are referred to here as general culturing conditions. Strain purity was confirmed using f/2 MM and f/2 MB purity test broths and visually confirmed by epifluorescence microscopy (73). Cultures (20 ml) were transferred weekly to maintain exponentially growing cultures.

Phytoplankton cells were enumerated by a hemocytometer or using a flow cytometer (Guava; Millipore). Via the flow cytometer, cell abundance was determined by using species-specific settings, including their forward scatter, side scatter, and red fluorescence (695/50-nm) emission characteristics for evaluating chlorophyll intensity. All samples were run at 0.24 μl s^−1^ for 3 min, either live or fixed with glutaraldehyde (0.5% final concentration). A correction factor was applied to fixed cell abundances to account for cell loss due to preservation.

### Growth experiments.

The HHQ concentration resulting in 50% growth inhibition (IC_50_) was determined using triplicate, 2- or 20-ml cultures of *E. huxleyi* (∼100,000 cells ml^−1^) exposed to HHQ (between 0.25 and 512 ng ml^−1^), PQS (0.5 to 530 μg ml^−1^), or the vehicle control (0.1% DMSO) for 72 h. Growth rates were calculated using an exponential growth equation and were plotted against the HHQ concentration to determine the IC_50_ at 72 h postexposure as described previously (16). Concentrations of DMSO below 0.5% (vol/vol) have no impact on axenic *E. huxleyi* growth. DMSO was used as the solvent vehicle for HHQ and PQS.

To examine the impacts of HHQ, triplicate flasks of 30-ml cultures of *E. huxleyi* (∼50,000 cells ml^−1^) were exposed to either 1 or 100 ng ml^−1^ HHQ or a vehicle (0.1% DMSO) control. The experiment mixture was sampled daily for 96 h to monitor *E. huxleyi* abundance, forward scatter, side scatter, red fluorescence (695/50 nm), and photosynthetic efficiency (*F_v_*/*F_m_*). *F_v_*/*F_m_* was measured using a fluorescence induction and relaxation (FIRe) system (Satlantic). Samples were dark adapted for 30 min, and photosystem II kinetics were measured from the average of 10 iterations of an 80-μs single-turnover event and 1,000 ms of weak modulated light.

To measure recovery, after 96 h of HHQ exposure, triplicate 2-ml aliquots of an HHQ-exposed culture were transferred into 198 ml of fresh medium, effectively diluting HHQ to 1 ng ml^−1^. The same dilution was made with the vehicle control treatment, and the experiment mixture was sampled daily for *E. huxleyi* growth rate, forward scatter, side scatter, and red fluorescence (695/50 nm).

To investigate viral infection dynamics, triplicate 50-ml cultures were prepared for the following treatments: *E. huxleyi* (∼40,000 cells ml^−1^) plus the vehicle control (0.1% DMSO), *E. huxleyi* plus EhV 207 (3.2 × 10^6^ EhV particles ml^−1^), *E. huxleyi* plus HHQ (100 ng ml^−1^), and *E. huxleyi* plus HHQ and EhV 207. The multiplicity of infection (MOI) was 80, to ensure successful viral infection potential. Samples were taken daily to monitor *E. huxleyi* abundance.

For all growth experiments, excluding the IC_50_ calculation, significant differences between treatments were determined by comparing abundances over time using ANOVAR, followed by Dunnett’s multiple-comparison test (74). All data were tested to ensure that they passed the assumptions for normality and sphericity prior to running the ANOVAR.

### Physiological assays.

Propidium iodide (PI) was used to quantitatively discriminate cell cycle stages in HHQ-exposed phytoplankton cultures over 122 h. Three replicate 2-liter cultures at ∼33,000 cells ml^−1^ were dosed with either 100 ng ml^−1^ HHQ or the vehicle control (0.002% DMSO). Fixed cells were enumerated every 24 h via flow cytometry. Every 2 h, approximately 10^6^ cells were subsampled, pelleted, and washed twice via centrifugation at 3,214 × *g* for 15 min at 18°C. The dry cell pellets were resuspended in 1 ml of ice-cold liquid chromatography-mass spectrometry (LC-MS)-grade methanol, transferred to microcentrifuge tubes, and stored at −80°C. For reading, methanol-fixed cells were centrifuged at 16,000 × *g* for 10 min at 4°C, methanol was removed, and pellets were resuspended in 1 ml of 1× Dulbecco’s phosphate-buffered saline (DPBS) before repelleting by centrifugation at 16,000 × *g* for 10 min at 4°C. The pellet was resuspended in 0.5 ml of FxCycle PI/RNase solution (Thermo Fisher), incubated for 30 min in the dark, and then measured via flow cytometry (583/26-nm emission).

Diagnostic fluorescent dye assays were used to measure indicators of cell stress and programmed cell death (PCD) following HHQ treatment. Intercellular reactive oxygen species (ROS) and nitric oxide (NO) production, mitotoxicity, cytotoxicity, and caspase protease levels and activity in whole-cell lysates were measured in *E. huxleyi* (starting cell concentration of ∼100,000 cells ml^−1^) following HHQ treatment (70 ng ml^−1^ or 100 ng ml^−1^) at various time points up to 72 h postexposure. See Text S1 in the supplemental material for detailed protocols.

10.1128/mSphere.00009-21.1 TEXT S1 Additional methodological information. Download Text S1, PDF file, 0.2 MB. Copyright © 2021 Pollara et al. 2021 Pollara et al. https://creativecommons.org/licenses/by/4.0/ This content is distributed under the terms of the Creative Commons Attribution 4.0 International license.

*E. huxleyi* DNA integrity was examined using a modified protocol for the Click-iT terminal deoxynucleotidyltransferase-mediated dUTP-biotin nick end labeling (TUNEL) Alexa Fluor 488 imaging assay kit (Thermo Fisher). Four replicate *E. huxleyi* cultures (∼250,000 cells ml^−1^) were assayed according to the manufacturer’s protocol and sampled after 46 h of HHQ exposure, with tagged cells being enumerated via flow cytometry (512/18-nm emission). See Text S1 in the supplemental material for detailed protocols.

### Transmission electron microscopy (TEM).

Replicate 20-ml cultures of exponentially growing *E. huxleyi* cells (∼100,000 cells ml^−1^) were exposed to either 100 ng ml^−1^ HHQ or the vehicle control (0.2% DMSO) for 24 h. Samples were concentrated by filtration on a 0.45-μm polycarbonate filter, transitioned out of f/2 medium via three sequential washes with 10 ml of 0.2 M sodium cacodylate buffer (pH 7.2), and then fixed in 2% glutaraldehyde in 0.2 M sodium cacodylate buffer (pH 7.2). Samples were postfixed in 2.0% osmium tetroxide for 1 h at room temperature and rinsed in double-distilled water (ddH_2_O) prior to *en bloc* staining with 2% uranyl acetate. After dehydration through a graded ethanol series, the cells were infiltrated and embedded in Embed-812 (Electron Microscopy Sciences). Thin sections were stained with uranyl acetate and lead citrate and examined with a JEOL 1010 electron microscope fitted with a Hamamatsu digital camera and AMT Advantage NanoSprint500 software.

### Transcriptomic and proteomic analyses.

A large-scale culturing experiment was performed with axenic *E. huxleyi* cells treated with either three concentrations of HHQ (1 ng ml^−1^, 10 ng ml^−1^, and 100 ng ml^−1^) or the vehicle control (0.002% DMSO) for 72 h. Following HHQ/DMSO exposure, 400-ml subsamples were taken from each quadruplicate 2-liter bottle at both 24 and 72 h for total RNA isolation, and an additional 1,200-ml subsample was taken at 72 h for total protein isolation. Total RNA and protein were isolated and quantified as described in Text S1 in the supplemental material.

For transcriptome sequencing (RNA-seq) analysis, the Kapa stranded mRNA-Seq library preparation kit (Kapa Biosystems) was used to prepare library samples, and the samples were sequenced on the NextSeq platform (Illumina) to generate 75-bp paired-end reads. Low-quality reads and adaptor sequences were trimmed using Trimmomatic (V0.38) (75). Transcript abundances were determined using Salmon (V0.12.0) (76) and the Ensembl (77) gene predictions for *E. huxleyi* CCMP1516 (the nonaxenic form of CCMP2090 [ftp://ftp.ensemblgenomes.org/pub/protists/release-41/fasta/emiliania_huxleyi/cdna/]) as a transcript target index (k-mer size = 23). Normalization and determination of significantly differentially abundant transcripts were performed using the DESeq2 R package (V1.22.1) (78). Tests for differential expression were carried out with the Wald test using a negative binomial generalized linear model. Logarithmic fold change (LFC) estimates were shrunken using the apeglm package (V1.6.0) (79) within DESeq2. The resulting *P* values were adjusted using the Benjamini-Hochberg (BH) procedure (80) (see Text S1 in the supplemental material).

For proteomic analysis, proteins were solubilized in urea, reduced, alkylated, and trypsin digested as described previously (81). The resulting peptide samples were desalted with a minicentrifugal C_18_ column according to the manufacturer’s instructions (Nest Group). Peptides were chromatographically separated (precolumn, 3-cm by 100-μm internal diameter [ID]; analytical column, 30-cm by 75-μm ID; resin, 3-μm C_18_-AQ) with a nanoAcquity ultraperformance liquid chromatography (UPLC) system (2 to 35% acetonitrile [ACN] and 0.1% [vol/vol] formic acid; 250 nl min^−1^ for 90 min) directly in line with a Fusion Lumos Orbitrap Tribrid mass spectrometer (Thermo Fisher Scientific) operated in the data-independent acquisition (DIA) mode according to methods described previously (82). To generate a peptide spectral library, 1 μg of a pooled sample containing equal parts from each peptide digest was analyzed with six gas-phase fractions covering *m/z* 400 to 1,000 in increments of 100 *m/z* (4 *m/z* staggered MS^2^ windows and 2 *m/z* overlap). Each bioreplicate was then quantified in single DIA analyses (MS^1^, *m/z* 400 to 1,000; 8 *m/z* staggered MS^2^ windows and 4 *m/z* overlap).

In order to generate absolute abundance measurements of detected proteins, raw MS data files were processed using msconvert (ProteoWizard) for demultiplexing and peak picking. EncyclopeDIA (V0.7.4) was used to (i) search the resulting fragmentation spectra against the UniProt *E. huxleyi* CCMP1516 protein and contaminant database (10.0-ppm precursor, fragment, and library tolerances), (ii) provide peptide-level area under the curve (AUC) data, and (iii) generate quantitative reports of identified peptides and proteins for each HHQ MS experiment (1% false discovery rate). Significant changes (*q *< 0.05) in protein abundances between HHQ treatments and the vehicle control were calculated as log_2_ fold changes between treatments. Complete details of protein sample preparations, chromatographic separations, mass spectrometry detection, and quantification can be found in Text S1 in the supplemental material.

Proteomic data were matched to the transcriptomic data utilizing the corresponding NCBI accession numbers. As many of the genes and proteins were uncharacterized, potential homologs of known proteins of interest were identified by querying the amino acid sequences of selected human proteins against the translated *E. huxleyi* (CCMP2090) genome, utilizing a significance threshold of an E value of <1 × 10^−20^. Combined data were visualized utilizing the ggplot2 and pheatmap packages in R.

### PARP inhibition and homology modeling.

To examine the impact of alkylquinolone exposure on mammalian PARP activity, an inhibition assay was performed using the PARP universal colorimetric assay kit (R&D Systems) according to the manufacturer’s instructions. Human PARP enzyme (0.5 U) was exposed to 50 μM HHQ (*n* = 4), 50 μM PQS (*n* = 4), or the vehicle control (0.25% DMSO) (*n* = 4) for 15 min prior to the addition of PARP activity buffer. See Text S1 in the supplemental material for a detailed protocol.

The *E. huxleyi* sequence under GenBank accession number XP_005783504.1 was aligned to the Protein Data Bank (PDB) database to determine the closest structural homolog with a small molecular inhibitor, veliparib, in the active site that could lend insight into HHQ binding.

### Detection of HHQ in environmental samples.

Seawater samples were collected along a cruise track from Manta, Ecuador, to Tahiti from October to December 2013 (U.S. GEOTRACES EPZT GP16) as described previously (83). Briefly, seawater was collected at a 3-m depth by a tow fish and pumped at a flow rate of 250 ml min^−1^ through a 0.2-μm filter and a polytetrafluoroethylene column packed with 20 g of polystyrene resin (Bondesil ENV; Agilent). Each sample represents an integrated average of 400 to 600 liters of water across a wide region. Samples were frozen onboard at −20°C. Prior to analysis, thawed columns were rinsed with 500 ml of 18.2 MΩ-cm ultrahigh-purity water (qH_2_O) and eluted with 250 ml of LC-MS-grade methanol. The extracts were concentrated by rotary evaporation and brought up in a final volume of 6 ml of qH_2_O that was stored at −20°C. The organic extracts were separated by a high-pressure liquid chromatography system (Dionex Ultimate 3000) coupled to an Orbitrap Fusion MS instrument (Thermo Scientific). The specific methodology can be found in Text S1 in the supplemental material.

### Data availability.

Sequences from this study have been deposited in the Gene Expression Omnibus (GEO) and are accessible through GEO series accession number GSE131846. The raw mass spectrometry proteomics data and subsequent spectral libraries have been deposited to the ProteomeXchange Consortium via the PRIDE partner repository under accession number PXD011560 (https://www.ebi.ac.uk/pride/archive/projects/PXD011560).
