# Supplementary figures and images for: Bacterial Quorum-Sensing Signal Arrests Phytoplankton Cell Division and Impacts Virus-Induced Mortality
Source: mSphere. 2021 May 12;6(3):e00009-21. doi: 10.1128/mSphere.00009-21 (PMC8125044; doi:10.1128/mSphere.00009-21)

**Supplemental Figure 1.**

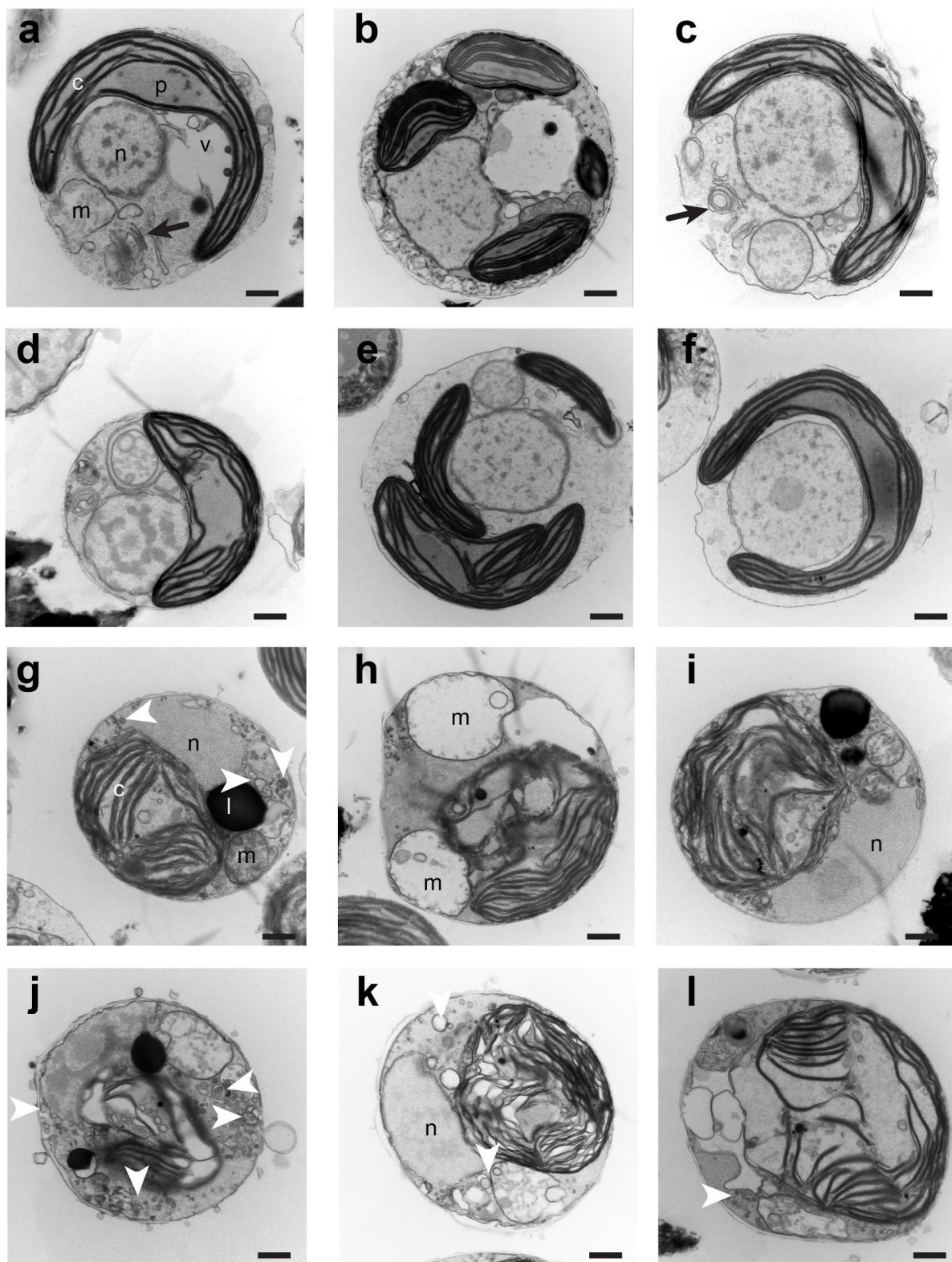

Supplement: FIG S1 [file mSphere.00009-21-sf001.pdf]

Supplemental Figure 2.

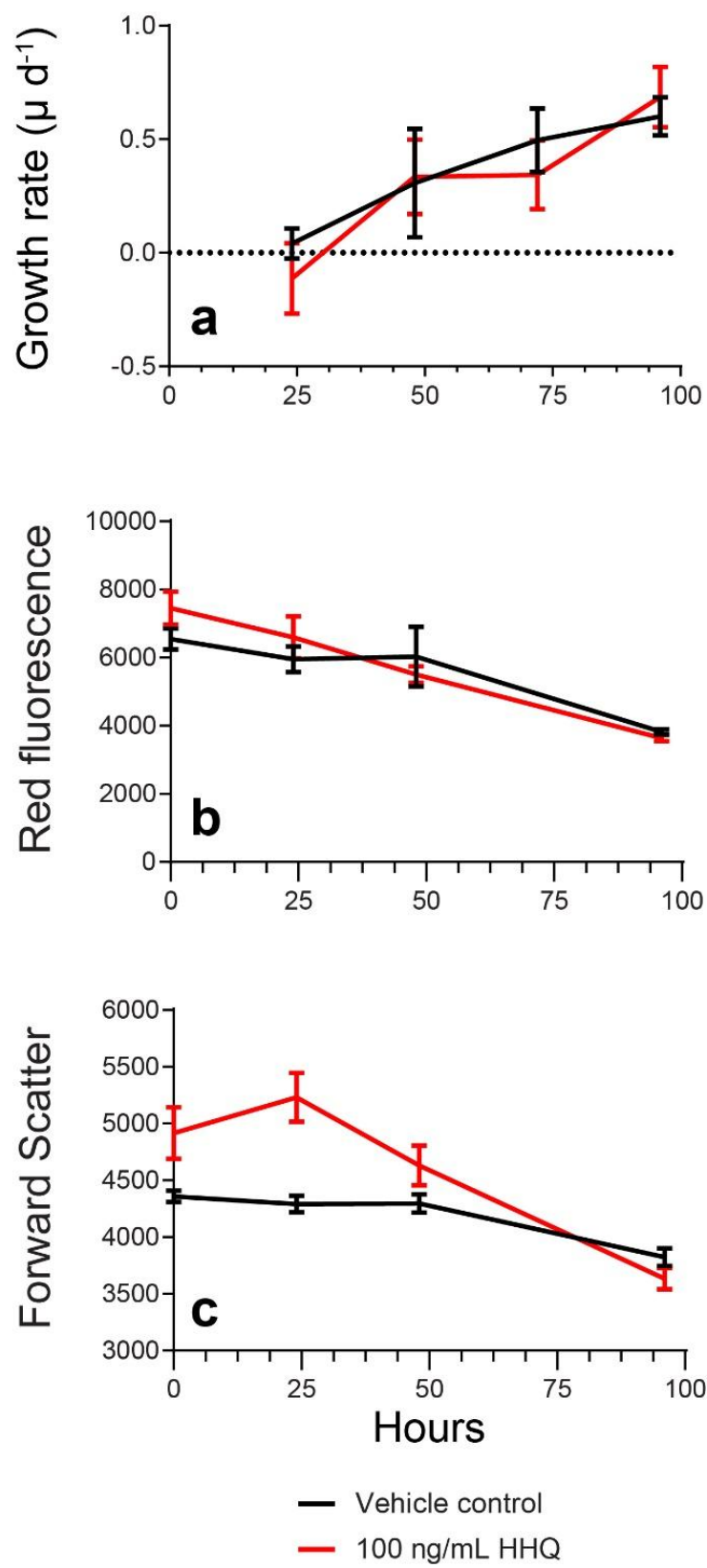

Supplement: FIG S2 [file mSphere.00009-21-sf002.pdf]

Supplemental Figure 3.

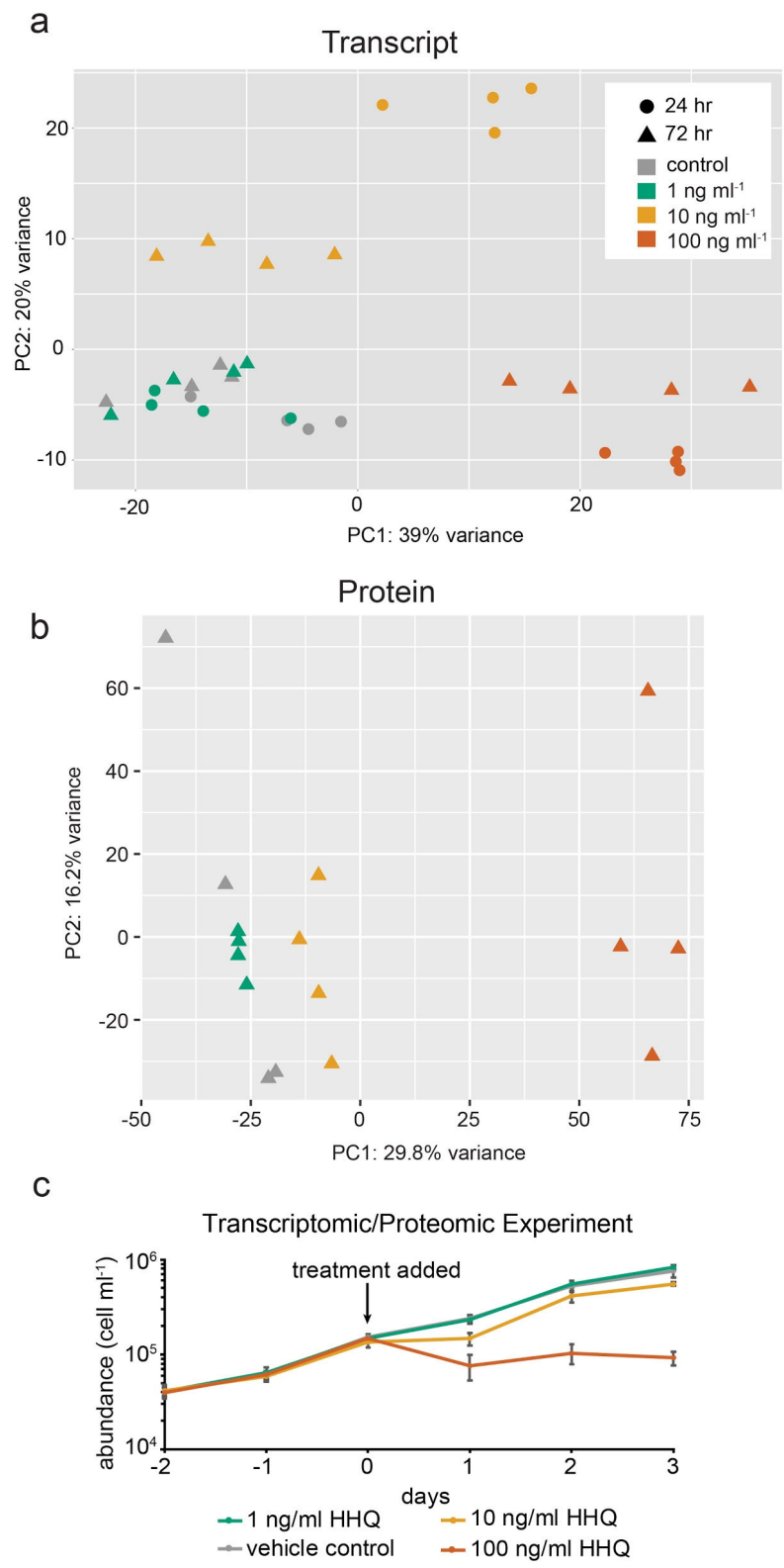

Supplement: FIG S3 [file mSphere.00009-21-sf003.pdf]

Supplemental Figure 4.

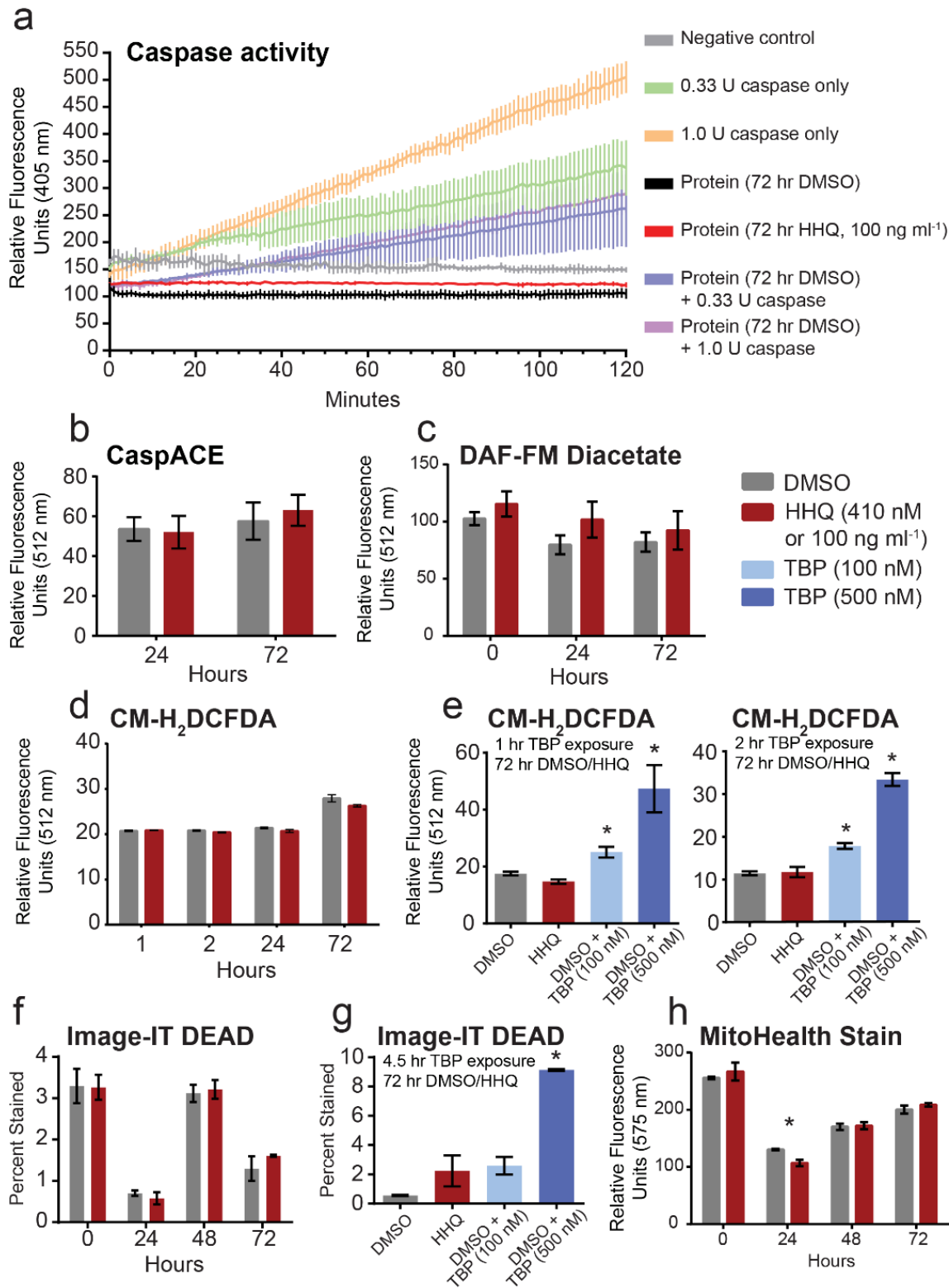

Supplement: FIG S4 [file mSphere.00009-21-sf004.pdf]

Supplemental Figure 6.

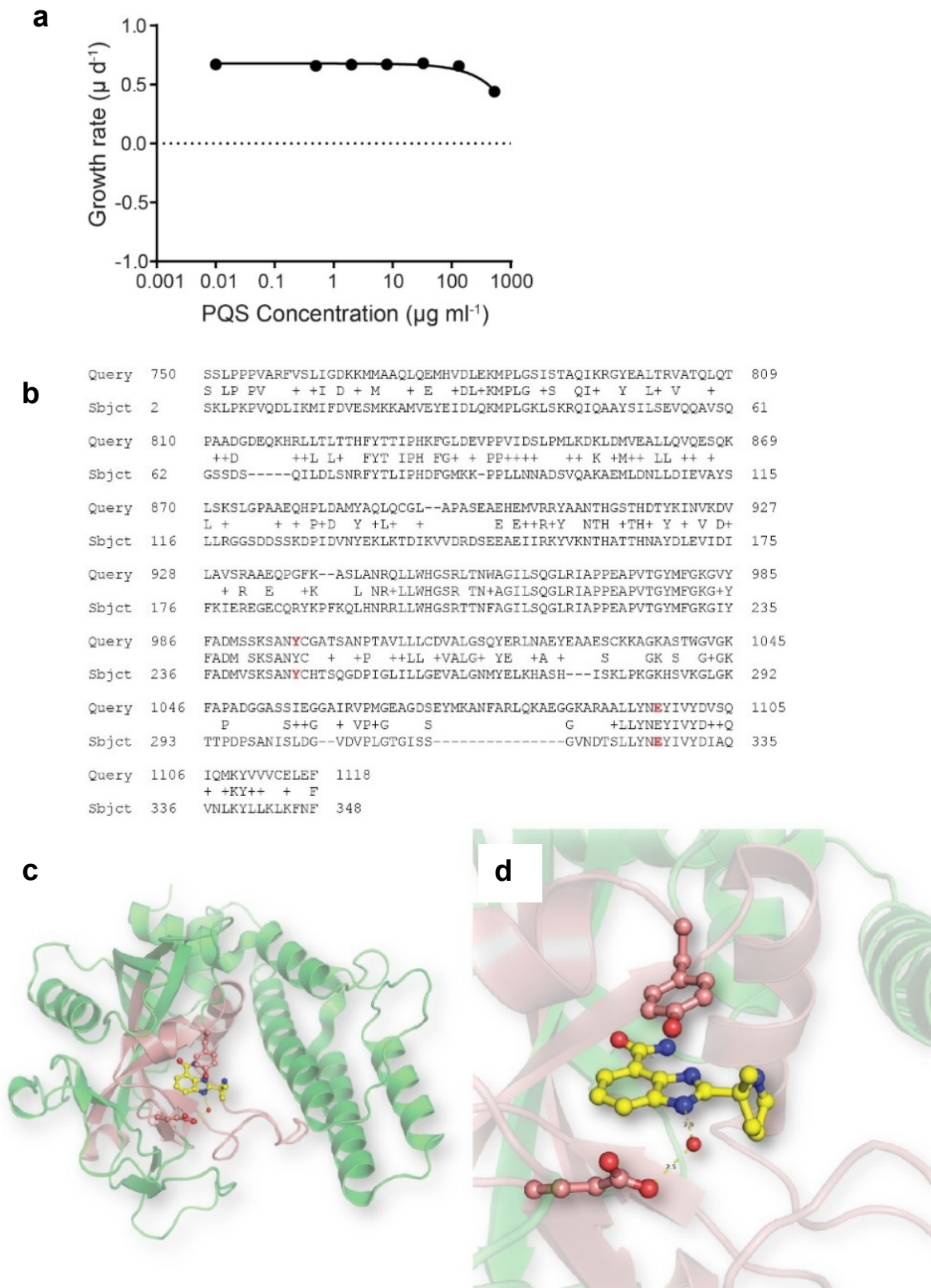

Supplement: FIG S6 [file mSphere.00009-21-sf006.pdf]

Supplemental Figure 7.

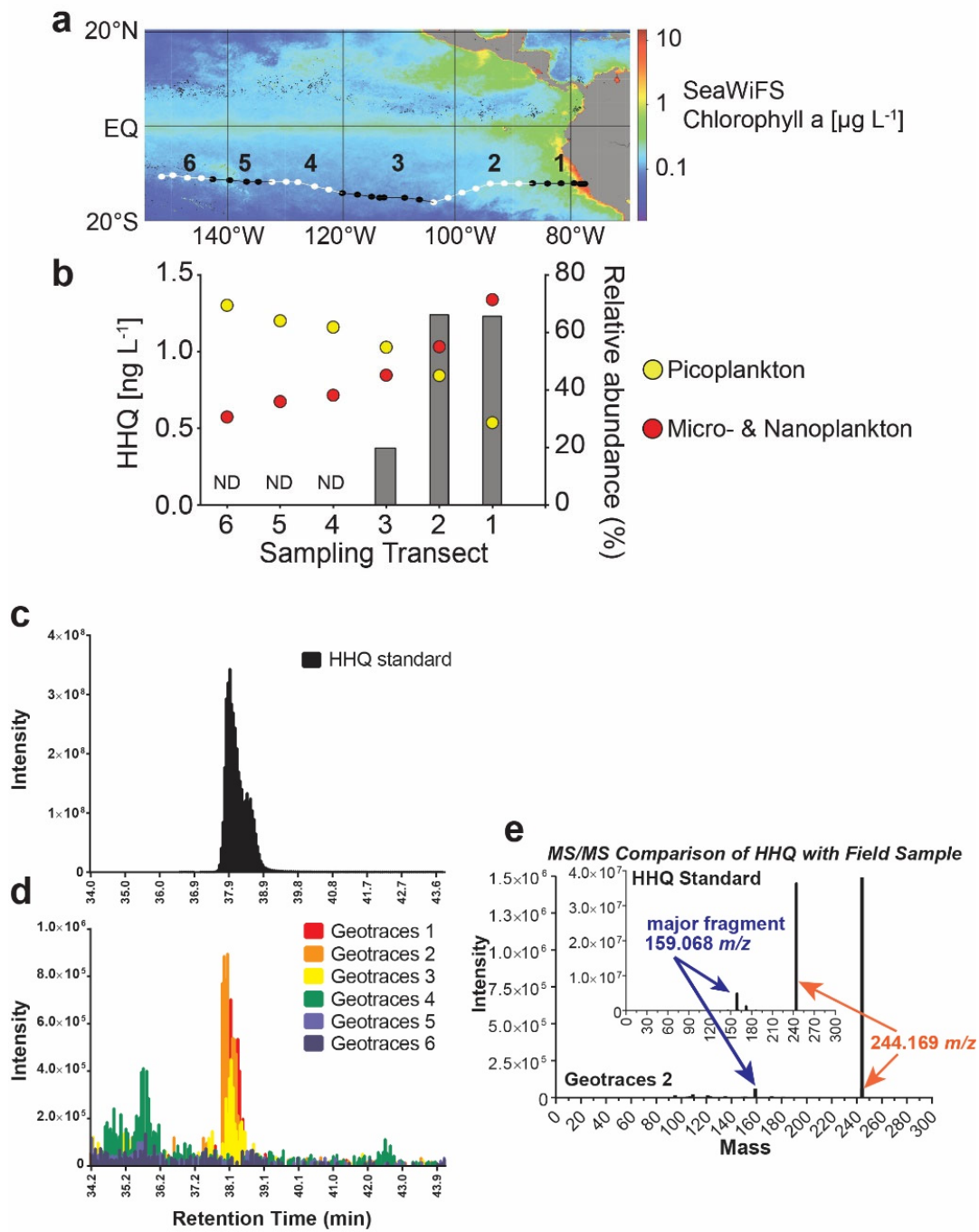

Supplement: FIG S7 [file mSphere.00009-21-sf007.pdf]
