# Supplementary material for: Bacterial Quorum-Sensing Signal Arrests Phytoplankton Cell Division and Impacts Virus-Induced Mortality
Source: mSphere. 2021 May 12;6(3):e00009-21. doi: 10.1128/mSphere.00009-21 (PMC8125044; doi:10.1128/mSphere.00009-21)
Supplement: FIG S5 [file mSphere.00009-21-sf005.pdf]

Supplemental Figure 5.

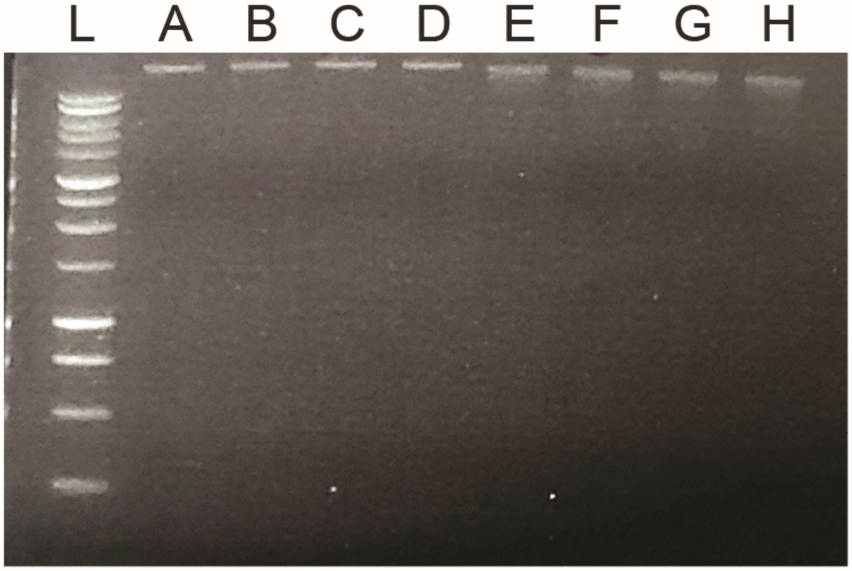

L: DNA ladder (Promega G5711)

A: 510 ng lambda + 1% DMSO (Rep 1)

B: 510 ng lambda + 1% DMSO (Rep 2)

C: 510 ng lambda + 100 ng/ml HHQ (Rep 1)

D: 510 ng lambda + 100 ng/ml HHQ (Rep 2)

E: 500 ng *E. huxleyi* 2090 gDNA + 1% DMSO (Rep 1)

F: 500 ng *E. huxleyi* 2090 gDNA + 1% DMSO (Rep 2)

G: 500 ng 2090 gDNA + 100 ng/ml HHQ (Rep 1)

H: 500 ng 2090 gDNA + 100 ng/ml HHQ (Rep 2)
