# Supplementary material for: Bacterial Quorum-Sensing Signal Arrests Phytoplankton Cell Division and Impacts Virus-Induced Mortality
Source: mSphere. 2021 May 12;6(3):e00009-21. doi: 10.1128/mSphere.00009-21 (PMC8125044; doi:10.1128/mSphere.00009-21)
Supplement: TEXT S1 [file mSphere.00009-21-sd001.pdf]

SUPPLEMENTAL INFORMATION TEXT

Bacterial quorum sensing signal arrests phytoplankton cell division and impacts virus-induced mortality

Scott B. Pollara[a], Jamie W. Becker[a,b], Brook L. Nunn[c], Rene Boiteau[d], Daniel Repeta[e], Miranda C. Mudge[c], Grayton Downing[a], Davis Chase[a], Elizabeth L. Harvey[f*], Kristen E. Whalen[a*]

[a]Department of Biology, Haverford College, Haverford, PA
[b]Present address: Science and Mathematics Department, Alvernia University, Reading, PA
[c]Department of Genome Sciences, University of Washington, Seattle, WA
[d]College of Earth, Ocean, and Atmospheric Sciences, Oregon State University, Corvallis, OR
[e]Marine Chemistry & Geochemistry, Woods Hole Oceanographic Institution, Woods Hole, MA
[f]Department of Biological Sciences, University of New Hampshire, Durham, NH

*Corresponding authors: kwhalen1@haverford.edu; elizabeth.harvey@unh.edu

Supplementary Information Text

Diagnostic biochemical assays assessing PCD/ROS response.

Cellular stress following HHQ treatment was examined via diagnostic fluorescent dye assays. Intracellular reactive oxygen species (ROS) production was measured by exposing 200 µl of exponentially growing *E. huxleyi* (2090) culture to 5 µM of CM-H_2DCFDA stain at 18 °C for 90 min in the dark. In triplicate, vehicle control or HHQ (100 ng ml^{-1}) was added to each sample well and ROS production was measured on the flow cytometer (Guava, Millipore) in 30 min intervals at 512/18 nm. *E. huxleyi* cultures exposed to 100 or 500 nM tetrabromopyrrole (TBP) for 1 and 2 hours served as a positive control for ROS production (1).

Intracellular nitric oxide production was measured by exposing 200 µl of exponentially growing *E. huxleyi* (2090) culture to 10 µM of DAF-FM diacetate at 18 °C for 60 min in the dark. In triplicate, vehicle control or HHQ (100 ng ml^{-1}) was added and NO production was immediately monitored on the flow cytometer at 512/18 nm. Additional cultures were monitored for the presence of NO at 24 and 72 h post exposure as described above.

Both mitotoxicity and cytotoxicity following HHQ exposure was assessed using the HCS Mitochondrial Health Kit (Thermofisher) according to manufacturer's instructions. The MitoHealth stain detects changes in mitochondrial membrane potential, whereupon the reagent accumulates in active mitochondria while the stain Image-iT® DEAD™ Green detects cytotoxicity. To 200 µl of an exponentially growing *E. huxleyi* (2090) culture, 80 µl of Cell Staining Solution containing MitoHealth Stain and Image-iT® DEAD™ Green was added to each sample and incubated in the dark at 18 °C for 30 min. Triplicate samples were dosed with vehicle control or 100 ng ml^{-1} HHQ and monitored at 512/18 nm and 575/25 nm on the flow cytometer for 24 h intervals. In a subsequent assay, the Image-iT® DEAD™ Green assay was repeated on triplicate *E. huxleyi* (2090) cultures exposed to vehicle control (DMSO) or 100 ng ml^{-1} HHQ for 72 h and either 100 nM or 500 nM of TBP for 4.5 h, used as a positive control, as described above.

The induction of active caspase proteases was assessed using the CaspACE™ FITC-VAD-FMK In Situ Marker (Promega). Single replicates of 20 ml *E. huxleyi* cultures (~25,000 cells ml^{-1}) were exposed to either vehicle control or 100 ng ml^{-1} HHQ. After 23 and 71 h, three technical replicates (400 µl) from each culture were collected and stained with 10 µM of CaspACE dye for 1 h at 18 ºC in the dark before monitoring at 512/18 nm via the flow cytometer.

For each of the experiments above, significant differences between treatments were determined by comparing abundances over time using a repeated measures analysis of variance (ANOVAR), followed by Dunnett's multiple comparisons test.

Caspase activity following HHQ exposure was measured using the Caspase-8/FLICE Fluorometric Assay kit (BioVision). Duplicate 2 L batch cultures were exposed to either 100 ng ml^{-1} HHQ or vehicle control (0.014% DMSO) for 72 h. At 72 h, 1.2 L of each culture was collected via centrifugation in acid-washed polypropylene bottles at 13,764 x *g* for 10 minutes at 4 °C to form a cell pellet that was transferred to a sterile 1.5 ml centrifuge tube. The pellet was centrifuged at 14,000 x *g* for 10 min at 4 °C. Supernatants were removed and dry cell pellets were flash frozen in liquid nitrogen prior to storage at -80 °C. Cell pellets were thawed on ice, resuspended in 1X Lysis Buffer (BioVision), lysed via sonication, and clarified via centrifugation at 12,000 x *g* for 10 min at 4 °C. Protein content of the clarified supernatants were measured using the Pierce Coomassie Plus Standard Microplate protocol (Thermo-Fisher).

Triplicate samples of 100 µg of each protein lysate were added to a black-sided, clear-bottomed microplate and volume was raised to 100 µl with additional lysis buffer. Triplicate wells were prepared with 100 µl of lysis buffer or lysis buffer containing varying concentrations of active Caspase-8 protein (0.33 U or 1 U), which served as negative and positive controls, respectively. The active Caspase-8 protein was also added in triplicate to 100 µg of protein lysate from DMSO-exposed cultures at final concentrations of 0.33 U and 1 U to demonstrate that pigments present in the protein lysates did not completely mask the fluorescent signal of the assay. To quantify caspase activity, 50 µM of IETD-AFC fluorogenic dye was added to each well. Fluorescence (Ex 400 nm, Em 505 nm) was collected every 1 min for 2 h via a Spectramax M2e plate reader (Molecular Devices) and HHQ versus vehicle control wells were compared at each time point using Welch's approximate t-test.

TUNEL assay.

E. huxleyi DNA integrity was examined using a modified protocol for the Click-iT TUNEL Alexa Fluor 488 Imaging Assay kit (Thermo Fisher). Replicate 20 ml cultures of exponentially growing *E. huxleyi* (~2 x 10^5 cells ml^{-1}) were exposed to either 100 ng ml^{-1} of HHQ (n = 4) or vehicle control (0.2% DMSO; n = 4). After 46 h, quadruplicate samples were collected from each culture by condensing ~500,000 cells on a 0.45 µm nylon filter, transitioned to 1X PBS, and fixed at 4% paraformaldehyde in 1X PBS for 45 mins in the dark. The fixative was removed by filtration and samples were resuspended in HPLC-grade methanol for 1 h. The methanol was removed and cells were washed with nuclease free water. Water was removed and all samples were pretreated with Terminal deoxynucleotidyl Transferase (TdT) reaction buffer for 10 minutes before incubation with the TUNEL reaction mixture containing TdT enzyme plus a dUTP label for 1 h at 37 ºC in the dark. The reaction mixture was removed and all samples were washed twice with 1X PBS supplemented with 3% Bovine Serum Albumin (BSA). Following the removal of the second wash, samples were incubated with Click-iT reaction cocktail for 30 min in the dark to attach the Alexa Fluor 488 tagged dUTP molecules. Samples were washed with 1X PBS with 3% BSA, resuspended in 1X PBS, and monitored for forward scatter, side scatter, and green (512/18 nm) fluorescence emission.

Direct assessment of DNA strand breaks.

To assess if HHQ directly is capable of inducing DNA strand breaks, duplicate samples of Lambda DNA (510 ng) and *E. huxleyi* (CCMP2090) genomic DNA (510 ng) were exposed to 100 ng ml^{-1} HHQ or DMSO vehicle control at 18°C for 24 h in the dark. Duplicate samples were applied to a 1% agarose gel and visualized by ethidium bromide.

Large scale culturing experiment for transcriptomic and proteomic analysis.

Four 2 L axenic *E. huxleyi* batch cultures in exponential phase were used to inoculate sixteen 2 L cultures at final cell concentrations of approximately 6.72 x 10^4 cells ml^{-1}. These cultures were grown for 48 h to approximately 1 x 10^5 cells ml^{-1}, before quadruplicate cultures were exposed to either 1 ng ml^{-1}, 10 ng ml^{-1}, or 100 ng ml^{-1} concentrations of HHQ or vehicle control (final concentration 0.002% DMSO in all bottles). Fixed cells were enumerated every 24 h by flow cytometry from all sixteen cultures. For all treatments, 400 ml subsamples were taken from each bottle at 72 h for protein isolation, and at both 24 and 72 h for total RNA isolation.

Biomass for isolation of total RNA was collected in acid-washed autoclaved polycarbonate bottles via centrifugation at 13,764 x *g* for 8 min at 4 ºC. Supernatant was

removed, and cell pellets were resuspended in 2 - 10 ml sterile seawater before aliquoting into microcentrifuge tubes (1 ml per tube). Biomass was again collected via centrifugation at 9,600 x *g* for 5 min at 4 ºC, the supernatant was removed, and pellets were flash frozen in liquid nitrogen prior to storage at -80 ºC. Total sampling time never exceeded 65 min per sample. Biomass for protein isolation was collected in acid-washed autoclaved polycarbonate bottles via centrifugation at 13,764 x *g* for 8 min at 4 ºC. Supernatant was removed, and cell pellets were resuspended in 20 - 30 ml sterile seawater before a second centrifugation at 1,500 x *g* for 8 min at 4 ºC. The majority of supernatant was removed before a final centrifugation at 1,500 x *g* for 5 min at 4 ºC and removal of all supernatant prior to flash freezing in liquid nitrogen and storage at -80 ºC. Total sampling time never exceeded 75 min per sample.

RNA processing and sequencing.

Total RNA was extracted using the RNeasy Plus Mini Kit (Qiagen) following the manufacturer's recommendations using 350 µl RLT plus buffer and the optional centrifugation step (18,800 x *g* for 1 min) prior to elution with 30 µl RNase free water. Eluent was reapplied to the column membrane and incubated for 8 min at room temperature before a second elution. Total RNA was quantified using a NanoDrop 2000 spectrophotometer (Thermo Scientific).

Total RNA for transcriptomic analysis was normalized to 20 ng μl^{-1} before assessing integrity using the Agilent 2100 Bioanalyzer System Plant RNA assay (Agilent Technologies). Samples were then prepared for sequencing at the Georgia Genomics and Bioinformatics Core facility using the KAPA Stranded mRNA-Seq library preparation kit with KAPA mRNA capture beads (Kapa Biosystems) and sequenced on the NextSeq platform (Illumina) to generate 75 bp paired-end reads. Data discussed in this publication have been deposited in NCBI's Gene Expression Omnibus - accessible through GEO Series accession number GSE131846 (http://www.ncbi.nlm.nih.gov/geo/query/GSE131846).

Sequence analysis.

Reads were conservatively trimmed to remove adaptors, low-complexity and low-quality sequence, and rRNA reads (including chloroplast and mitochondria rRNA) using Trimmomatic (V0.38) with a custom adapter file and the following settings: ILLUMINACLIP:2:30:10 LEADING:3 TRAILING:3 MAXINFO:40:0.5 MINLEN:50. Read quality was examined before and after trimming using FastQC (V0.11.8; https://www.bioinformatics.babraham.ac.uk/projects/fastqc/) and MultiQC (V1.6; (2)). Salmon tool (V0.12.0) was used to determine transcript abundances in quasi-mapping mode with default settings and the following flags: --validateMappings; –gcBias. Quantification results were examined using MultiQC and processed using tximport (V1.10.0; (3)). Transcript and gene IDs were linked using the general feature format file for *Emiliania huxleyi* CCMP1516 (ftp://ftp.ensemblgenomes.org/pub/protists/release-41/gff3/emiliania_huxleyi). After estimation of size factors to normalize for differences in library sequencing depth and gene dispersion estimation, tests for differential expression were carried out using DESeq2 (V1.22.1) for each pairwise comparison of interest. Data visualization, including principal component analyses, were performed directly within the DESeq2 package after the data set was made homoskedastic by a regularized-logarithm transformation.

Protein extraction and detection.

Frozen cell pellets were thawed on ice and resuspended in 6 M urea in 4 ºC 50 mM ammonium bicarbonate buffer. Cells were lysed using a sonicating probe for 10 rounds (4 ºC, 10 s, 0.7-0.9 W) with 30 s rests between rounds. Lysate was centrifuged (16,000 x g for 10 min, 4 °C) and protein content of supernatant was determined using the colorimetric bicinchoninic acid assay (BCA) assay (Thermo Fisher) following manufacturer's instructions for a 96-well plate on a Spectramax 190 microplate reader (Molecular Devices).

To digest proteins, 100 µg of protein lysate in 100 µl with 6 M urea in 50 mM ammonium bicarbonate buffer was reduced using Tris (2-carboxyethyl) phosphine hydrochloride (TCEP; 2.5 µl of 200 mM) after the addition of tris buffer (pH 8.8) (6.6 µl of 1.5 M stock) to maintain a pH of 8 prior to incubating the sample for 1 h at 37 °C. Samples were then reduced with iodoacetamide (IAA) (20 µl of 200 mM stock; 1 h, 20 ºC, in dark) and remaining IAA was quenched with dithiothreitol (DTT; 20 µl of 200 mM;1 h, 20 ºC, in dark). Samples were diluted with 800 µl of 25 mM ammonium bicarbonate buffer and 200 µl HPLC grade methanol. Proteins were digested with 5 µg trypsin (12 h, 20 ºC, in dark). Samples were acidified with 30-40 µl of 50 % formic acid to pH < 2 before evaporating to dryness in a centrivacuum. Peptide samples were stored at -80 °C until analysis.

Peptide samples were resuspended in 2% ACN and 0.1% formic acid and desalted using centrifugal BioPure SPN MIDI PROTO solid phase extraction C18 column (PROTO, 300Å; Nest Group) following manufacturer's instructions. Final peptide samples were evaporated to dryness using a speedvac and resuspended in 100 µl of 2% ACN and 0.1% formic acid to final concentrations of 1 µg μl^{-1}. Individual sample vials were prepared by mixing 10 µl of peptide sample with 1 µl of 500 fmol μl^{-1} Peptide Retention Time Calibration (PRTC; ThermoFisher Scientific) and 19 µl of 2% ACN and 0.1% formic acid. A pooled peptide sample was prepared by combining 3 µl from each individual sample vial and mixed by vortexing. Quality control (QC) samples (ThermoFisher Scientific) were prepared by mixing 10 µl of PRTC (Pierce Peptide Retention Time Calibration Mixture; Thermofisher) with 30 µl of 0.2% ACN, resulting in a final solution of 50 fmol μl^{-1} PRTC.

Five QC samples were analyzed to ensure chromatographic reproducibility and ion intensity stability before starting sample data collection. For each sample analysis, 1 µg of sample peptides with 50 fmol of PRTC were chromatographically separated by reverse-phase chromatography using a 30 cm long, 75 µm i.d., fused silica capillary column (New Objective PicoTip) packed with C18 beads (Reprosil-Pur C18-AQ 3 µm (Dr. Maisch GmBH, Ammerbuch, Germany) in line after a with a 3 cm long, 100 µm i.d. precolumn (C18-AQ 3 µm Dr. Maisch GmBH). Peptides were eluted using a linear acidified (formic acid, 0.1% v/v) water-acetonitrile gradient (2–35% acetonitrile; 90 minute). Mass spectrometry (MS) was performed on the Lumos, operated in data independent acquisition mode (DIA) The pooled peptide sample was analyzed using 6 narrow-window (100 m/z each) gas phase fractionations (GPFs) spanning 400-1000 m/z with 4 m/z overlapping MS2 windows with mass spectrometry settings set to 120,000 precursor resolution and 30,000 fragment resolution, automatic gain control (AGC) target of 4e5, 60 ms maximum ion inject time (IIT), Normalized Collision Energy (NCE) of 33, and +2H default charge state. Each MS2 window for the pooled sample experiments overlapped with the next window by 2 m/z, proving high resolution and effectively duplicate 2 m/z sampling throughout. The pooled sample was used to generate a complete peptide spectral library for *E. huxleyi*. For individual sample quantification, DIA data was collected on ions 400-1000 m/z using 8 m/z staggered MS2 windows with MS settings of 120,000 precursor resolution, 15,000 fragment resolution, AGC target of 4e5, max IIT of 20 ms. Each MS2 window for the individual sample

DIA experiments overlapped with the adjacent window by 4 m/z. The order of the MS experiments was randomized, and the first sample was re-injected after all other experiments were completed to determine if a chromatography shift had occurred. Quality control (QC) peptide mixtures (Pierce mixed peptide PRTC standards) were analyzed every 12th injection to monitor chromatography and MS sensitivity. Skyline was used to determine that QC standard retention time and isotopic distribution did not deviate > 10% through all analyses.

Proteomic data analysis.

All MS data was interrogated against a peptide database generated from *E. huxleyi* CCMP1516 genome (downloaded from Uniprot; downloaded Oct. 5, 2018) appended with a list of 50 common contaminant peptides and the QC standards.[1] All raw MS data was first demultiplexed by msconvert (ProteoWizard). A chromatogram library was generated from the pooled sample analyses using XCorDIA software in EncyclopeDIA 0.7.4. This analysis on the pooled samples provided peptide detection, fragment refinement, and determination of peak boundaries, generating a library of all detected peptides with associated peak area under the curve (AUC) values (a measure of absolute abundance). The chromatogram library ELIB file was generated in XCorDIA by processing mzML pooled files as "Jobs" against the background *E. huxleyi* FASTA proteome (35,707 proteins). Parameters adjusted based on experiment-specific details with the Data Acquisition Type set as "Non-Overlapping DIA." EncyclopeDIA was then used to search the wide-window individual sample data with the same "Background" UniProt FASTA for *E. huxleyi*. mzML files of individual samples were loaded as "Jobs" and processed with the same parameters as set for XCorDIA to generate quantitative reports, BLIB files, and a comprehensive chromatogram library. Detailed settings included: Target/Decoy Approach: normal; Data Acquisition Type: Non-overlapping DIA; Enzyme: Trypsin; Fixed: C+57 (Carbamidomethyl); Variable: None; Fragmentation: CID/HCD (B/Y); Precursor Mass Tolerance: 10.0 ppm; Fragment Mass Tolerance: 10.0 ppm; Maximum Missed Cleavage: 2; Percolator Version: v3-01; Percolator FDR threshold: 0.01; # Quantitative Ions: 5; Minimum # Quantitative Ions: 1; # Cores: 8; Charge range: 1 to 4. Quantitative reports of the detected proteins and the absolute abundance of these proteins were generated.

Principal component (PC) analysis of AUC data was performed by adding a value of 1 to each peak area value in the quantitative report, completing a logarithmic transformation of the data, and generating a principal component plot of PC1 and PC2 (ggbiplot package in R).

To examine the differential expression in each treatment, normality was confirmed visually and with the Shapiro-Wilk test. Equality of variance between the AUC values of HHQ treatments could not be determined via the F-test. The F-test indicated that 445 proteins (8% of total proteins detected) in the 100 ng ml^{-1}, 328 proteins (6%) in the 10 ng ml^{-1}, and 432 (8%) in the 1 ng ml^{-1} HHQ treatments had significantly unequal variances. Welch's approximate t-test was therefore used to compare the mean AUC of proteins in each HHQ treatment (1-, 10-, 100 ng ml^{-1}) to the mean AUC of the DMSO control proteins. Differentially expressed proteins were determined using two levels of significance (adj. $p < 0.1$ and adj. $p < 0.05$) to stay consistent with the analysis of the transcriptomic data.

The change in protein expression level between each treatment and the DMSO control were calculated as log2 fold-change using the equation below (i.e., value of 1 is a 2-fold

---

[1] Common contaminants in proteomics include proteins present in dust such as human or animal keratin.

difference, while a value of 3 is an 8-fold difference). Negative values represent decreased abundance of proteins in the treatment relative to DMSO control, while positive values represent increased abundance in the treatment relative to DMSO control.

$$log_2\left(\frac{Mean\ Peak\ Area\ of\ Treatment}{Mean\ Peak\ Area\ of\ Control}\right) = FC$$

Lists of all GO identification codes associated with various gene/protein functions were generated by searching the cellular component, biological process, and molecular function GO hierarchies for key words associated with selected pathways (see Supplemental Data File 1 at https://doi.org/10.6084/m9.figshare.14414285.v1) (4). For each pathway, a specificity threshold ID was manually chosen (see Supplemental Data File 1 at https://doi.org/10.6084/m9.figshare.14414285.v1) and all GO IDs below the threshold were compiled. These lists of GO IDs were then used to color the most stringent expression level scatterplot (adj. $p < 0.05$) by pathway; GO terms were compiled and used to color the scatterplot. The NCBI Protein BLAST tool was used to compare the amino acid sequence of selected *E. huxleyi* proteins to better annotated translated genomes of other organisms (5). The BLAST results (significance threshold: e-value < 1×10^{-20}) were used to determine the likely identity of these selected proteins through sequence homology and to validate the biological category associated with each list of proteins.

PARP inhibition.

To examine the impact of alkyl quinolone exposure on mammalian poly(ADP-ribose) polymerase (PARP) activity, an inhibition assay was performed using the PARP Universal Colorimetric Assay Kit (R&D systems). In a histone-coated strip-well plate, duplicate wells without inhibitor or without enzyme served as positive and negative controls, respectively. Four replicates of 0.5 U of mammalian PARP enzyme was pre-incubated with 50 µM HHQ, 50 µM PQS, or vehicle control (0.25% DMSO) for 15 min prior to the addition of a PARP activity buffer containing biotinylated-nicotinamide and activated DNA for 1 h in the dark. Wells were washed twice with 200 µL of 1X PBS + 0.1% Triton X-100, followed by two additional washes with 1X PBS before the addition of Strep-HRP for 1 h in the dark. The wells were washed as before and 50 µl of pre-warmed TACS-Sapphire colorimetric substrate was incubated for 15 minutes in the dark. The reaction was stopped and color stabilized by the addition of 50 µl of 0.2 M HCl. Absorbance at 450 nm was measured using Tecan Infinite M200 plate reader.

Detection of HHQ in environmental samples.

Organic seawater extracts were separated by high pressure liquid chromatography (Dionex Ultimate 3000) coupled to an Orbitrap Fusion MS (Thermo Scientific). Separations were performed on 50 µL injections on a C8 column (2.1 × 100 mm, 3 µm particle size; Hamilton) using a 50 min gradient from 10% to 90% methanol in water with 5 mM ammonium formate (LCMS grade, Sigma-Aldrich) as a buffer. The orbitrap was equipped with a heated electrospray ionization source with a capillary voltage of 3500 V; sheath, auxiliary, and sweep gas flow rates of 12, 6, and 2 (arbitrary units); and ion transfer tube and vaporizer temperatures of 300 and 75 °C. Mass spectra were collected in positive mode at 450K resolution. Fragmentation spectra were collected using a quadrupole isolation window of 1 m/z and high-

energy collision-induced dissociation (HCD) energy of 35%. The limit of detection for HHQ using the described method was 0.18 ng L^{-1}.

References

1. K. E. Whalen *et al.*, The chemical cue tetrabromopyrrole induces rapid cellular stress and mortality in phytoplankton. *Sci Rep* **8**, 15498 (2018).
2. P. Ewels, M. Magnusson, S. Lundin, M. Kaller, MultiQC: summarize analysis results for multiple tools and samples in a single report. *Bioinformatics* **32**, 3047-3048 (2016).
3. C. Soneson, M. I. Love, M. D. Robinson, Differential analyses for RNA-seq: transcript-level estimates improve gene-level inferences. *F1000Res* **4**, 1521 (2015).
4. M. Kanehisa, M. Furumichi, M. Tanabe, Y. Sato, K. Morishima, KEGG: new perspectives on genomes, pathways, diseases and drugs. *Nucleic Acids Res* **45**, D353-D361 (2017).
5. J. Gough, K. Karplus, R. Hughey, C. Chothia, Assignment of homology to genome sequences using a library of hidden Markov models that represent all proteins of known structure.
